# KDM4C works in concert with GATA1 to regulate heme metabolism in head and neck squamous cell carcinoma

**DOI:** 10.1007/s00018-025-05693-x

**Published:** 2025-04-21

**Authors:** Meng-Jen Wu, Shan-Min Yang, Wei-Kai Fang, Tsan-Jan Chen, Chun-Yi Wu, Yen-Jung Hsu, Cheng-En Shen, Yu-Chia Cheng, Wan-Chen Hsieh, Chiou-Hwa Yuh, Muh-Hwa Yang, Hsing-Jien Kung, Wen-Ching Wang

**Affiliations:** 1 Institute of Molecular and Cellular Biology and Department of Life Science, National Tsing-Hua University, Hsinchu, 30013 Taiwan, ROC; 2 Institute of Molecular and Genomic Medicine, National Health Research Institutes, Miaoli, 35053 Taiwan, ROC; 3 Institute of Clinical Medicine, National Yang Ming Chiao Tung University, Taipei, 11221 Taiwan, ROC; 4 Graduate Institute of Cancer Biology and Drug Discovery, Taipei Medical University, Taipei, 11031 Taiwan, ROC; 5 Department of Biochemistry and Molecular Medicine, University of California Davis School of Medicine, University of California Davis Cancer Centre, Sacramento, CA 95817 USA

**Keywords:** Head and neck squamous cell carcinoma, KDM4C, GATA1, Histone demethylase, Heme metabolism, FECH

## Abstract

Head and neck squamous cell carcinoma (HNSCC), the sixth most common cancer worldwide, presents significant public health challenges due to its genetic instability and late-stage diagnosis. Despite advancements in treatment, the median overall survival remains below one year, emphasizing the need for improved detection, prognosis, and therapeutic strategies. This study investigates the role of KDM4C and its interaction with GATA1 in regulating heme metabolism and tumor progression in HNSCC. KDM4C knockdown (KDM4C-KD) hindered HNSCC cell migration using *in vitro* assays, inhibited metastasis through zebrafish xenotransplantation, and suppressed tumor growth in mouse xenograft models. RNA-seq and CUT&Tag-seq analyses on KDM4C-KD SAS cells identified KDM4C-regulated genes, including ferrochelatase (FECH), in heme metabolism. Immunoprecipitation and docking analyses confirmed the KDM4C-GATA1 interaction. Notably, FECH overexpression in KDM4C or GATA1 knockdown cells restored cell migration, invasion, and proliferation, highlighting FECH as a crucial downstream target. KDM4 inhibitors myricetin and BPRKD022S0 (22S0) increased H3K9me3 levels, downregulated heme metabolism genes, and reduced cell survival in HNSCC cells. Zebrafish and mouse models demonstrated that these inhibitors effectively suppressed tumor growth and metastasis. Immunohistochemical analysis of HNSCC patient samples revealed high KDM4C and GATA1 expression correlated with advanced clinical stages and poor survival outcomes. Our findings elucidate the critical role of the KDM4C/GATA1-FECH axis in HNSCC progression and suggest that targeting this pathway with KDM4 inhibitors shows promising therapeutic potential for HNSCC treatment.

**Supplementary Information:**

The online version contains supplementary material available at 10.1007/s00018-025-05693-x.

## Introduction

Head and neck squamous cell carcinoma (HNSCC), ranking as the sixth most common cancer globally, presents a significant challenge to public health [1]. This malignancy is characterized by its genetic instability and typically progresses from epithelial cell hyperplasia to invasive carcinoma. A major hurdle in managing HNSCC is its late diagnosis, often at an advanced stage. The primary mode of treatment failure is local-regional recurrence [2]. Even with advancements in treatments, the median overall survival for HNSCC patients remains low, less than one year, highlighting an urgent need for detection, prognosis, and improved therapeutic strategies [3, 4].

Hanahan and Weinberg have significantly contributed to our understanding of the processes involved in cancer development and progression [5, 6]. One important pathway leading to cancer development is epigenetics, which involves inheritable changes in the expression of genes without DNA sequence alterations. These changes play a crucial role in the initiation and progression of cancer. Modifications of the proteins that package DNA, such as acetylation, methylation, and phosphorylation of histones, can impact gene expression [7, 8]. Histone lysine demethylase (KDM) members are a group of enzymes that can regulate gene expression through epigenetic mechanisms [9–11]. They do this by removing methyl groups from histone lysine, which alters chromatin structure and affects gene expression. The relationship between KDMs and cancer is complex, as these enzymes can either suppress or promote the development and progression of cancer in a context-dependent manner [12–14].

Unique metabolic adaptations of cancer cells also play an essential role in supporting rapid tumor growth and spread. Heme metabolism has emerged as a crucial process in this context, significantly influencing cancer progression and metastasis [15–17]. Heme, an essential iron-containing cofactor for various aerobic processes, is primarily synthesized in the mitochondria [18]. Cancer cells frequently exhibit increased heme production, which fuels their aggressive behavior through enhanced mitochondrial respiration [19–21]. This elevated heme production not only supplies prosthetic groups for the oxidative phosphorylation complexes, crucial for efficient energy production [22] but also leads to increased ATP demand and oxygen consumption [17, 20]. As a result, a hypoxic environment is created within the tumor microenvironment (TME). Hypoxia then activates various pathways, including hypoxia-inducible factor-1 and vascular endothelial growth factor, which contribute to dysfunctional vascularization, tumor invasion, metastasis, and therapeutic resistance [23]. The hypoxia present in the TME results in the formation of an immunosuppressive environment, further complicating treatment outcomes [23]. These insights into heme metabolism suggest its complex role in the metabolic reprogramming of cancer cells and its significant impact on tumor progression and treatment efficacy.

In this study, we assessed the clinical relevance of KDMs in HNSCC using Kaplan-Meier plotter online tool and TCGA analysis. Our findings indicate that KDM4C, a demethylase that removes the methyl group from H3K9me3/2 and H3K36me3/2 through its JmjC-domain [24, 25], is overexpressed and is associated with poor clinical outcomes. We showed that KDM4C acts as a coactivator of GATA1, which is a known transcription factor involved in hematopoiesis [26], and regulates the expression of heme-metabolism genes by demethylating H3K9me3.

Interestingly, KDM4C has been reported to be overexpressed in multiple cancer types, including breast and prostate cancers, where it plays a key role in promoting cell proliferation, gene regulation, and tumor progression [27, 28]. Similarly, GATA1 is known to play pivotal roles in hematologic cancers, such as acute erythroid leukemia, and recent studies indicate that it may also hold regulatory functions in solid tumors [29, 30]. While studies have shed light on the roles of KDM4C and GATA1 in gene regulation and hematologic diseases, the specific interplay between these proteins in heme metabolism remains unexplored. Moreover, little is known about how this interaction influences heme metabolism and impacts tumor progression and metastasis.

Our study addresses this gap by examining how the interaction between KDM4C and GATA1 collaboratively regulates FECH expression, providing new insights into the molecular mechanisms linking heme metabolism to tumor growth and aggressiveness. We further demonstrate that Inhibiting KDM4C reduces cancer progression, as validated in zebrafish xenotransplantation and mouse graft models. These findings establish the KDM4C-GATA1 axis as a critical driver of HNSCC malignancy and suggest that KDM4C is a promising therapeutic target for intervention in HNSCC and potentially other cancers regulated by this pathway.

## Materials and methods

### Cell culture

The human HNSCC cell lines (SAS and FaDu) and a human embryonic kidney cell line HEK293T originally from ATCC were used in this study. The SAS-LN cells have been previously described [31]. SAS, SAS-LN, SG and HEK293T cultured in Dulbecco’s Modified Eagle’s medium (DMEM) (Gibco) supplemented with 10% fetal bovine serum (FBS) (Gibco). FaDu cultured in RPMI-1640 (Gibco) supplemented with 10% fetal bovine serum (FBS). Cells were incubated at 37 ℃, 5% CO_2_ atmosphere.

### RNA interference

To generate recombinant lentivirus, HEK293T cells were transfected with a lentiviral vector encoding pLKO.1 or shRNA targeting *KDM4C*, *GATA1*, and *FECH* genes. PolyJet (SignaGen) in vitro DNA transfection reagent was used for transfection. Packaging vectors pLP1, pLP2, and pLP/VSVG were from Thermo. After 76 hours, virus particles were harvested from the supernatant. HNSCC cells were then transduced with pLKO.1 or shRNA viral particle, followed by 48 hours of selection with puromycin. The knockdown efficiency was confirmed by using Western blotting analysis and qPCR assay. For siRNA transfection, experiments followed the protocol of Lipofectamine 3000 (ThermoFisher).

### Antibodies, plasmids, and reagents

All antibodies used in this study are described in Table S1. For RNA interference, the source of plasmids and target sequence are described in Table S2. The pCMV-HA-KDM4C (Cat. No. 24214) and the pcDNA3.1-myc-GATA1 (Cat. No. 118352) expression plasmids were purchased from Addgene. The various truncated forms of KDM4C (ΔN135, ΔN490, and ΔC185) or GATA1 (∆C170, ∆N199 and ∆N249) were generated by PCR and cloned to pCDNA3.1-HA or pCDNA3.1-myc, respectively. The FECH cloning plasmid was purchased from Sino Biological (Cat. No. HG17103-U), then amplified and cloned to pCDNA3.1-Flag. All cloning primers used in this study are described in Table S3.

### Immunoblotting

To obtain total cell lysates, the cells were lysed with RIPA buffer supplemented with protease inhibitor (Roche). Next, the lysates were separated by SDS-PAGE on a 10% acrylamide gel and then transferred onto a nitrocellulose blotting membrane (Cytiva) membrane with 0.45 µm pore size. The membrane was then incubated with diluted primary antibodies at 4 °C overnight. Following this, the membrane was probed with secondary antibodies conjugated with horseradish peroxidase (HRP) for 4 hours at room temperature. The reagent kit, Trident femto Western HRP Substrate (GenTex, GTX14698), was used for chemiluminescence generation. Finally, the level of signals was detected using the iBright Imaging System (Thermo).

### Wound healing and invasion assays

HNSCC cells were plated in 6-well plates and incubated overnight until they reached a density of approximately 90% as a monolayer. A cross-shaped wound was created in the cell monolayer using a 10-μL pipette tip, and the wound was monitored at specific time points using a Leica microscope. The area of wound closure was quantified using MRI_Wound_Healing_Tool of ImageJ software.

### Invasion assay

The invasion assay followed BD BioCoat Matrigel invasion Chamber (BD Biosciences, Cat. No. 354480) guidelines. Briefly, 7×10^4^ cells in 500 μL serum-free culture medium were plated onto the upper chamber, and 10% FBS was added to the lower chamber. Cells were incubated at 37 ℃ for 24 hours. After incubation, the invading cells that transited to the lower membrane were fixed with 3.7% paraformaldehyde (Thermo Scientific) and stained with crystal violet. The non-invading cells were scrubbed with cotton swabs. For quantification, the crystal violet extraction buffer (50% EtOH, 0.1% acetic acid) was used for extraction, and the absorbance was detected at OD570 nm by CLARIOstar® Microplate Reader (BMG Labtech, Ortenberg, Germany).

### Quantitative RT-PCR (qRT-PCR)

Cells were collected with TRIzol reagent (Invitrogen), separated by chloroform, precipitated by isopropanol, and dehydrated by ethanol. The concentration of total RNA was measured using Nano-DropTM Spectrophotometers (Thermo). Appropriate amounts of total RNA were reverse transcribed using the ReverTra Ace Set (Purigo). The SensiMixTM SYBR® Hi-ROX Kit from Bioline (Taunton, MA, USA) and primers (Supplementary Table S5) were used to perform PCR. 18S was used as an internal control. The ABI StepOnePlus Real-Time PCR System (Thermo) was utilized for the PCR process.

### RNA-seq

Control (LKO) and shKDM4C SAS cells (1×10^6^ cells) were seeded in a 10-cm dish and incubated at 37 °C overnight. Total RNA extraction was described previously. The samples were then sent to Biotools Co., Ltd. in Taiwan (R.O.C) for quality control, NGS service, and data analysis assistance. The sequencing libraries were prepared using the KAPA mRNA HyperPrep Kit (KAPA Biosystems, Roche, Basel, Switzerland) according to the manufacturer's instructions. High-throughput sequencing was conducted on the Illumina NovaSeq 6000 platform, and the raw sequenced reads were generated using CASAVA base calling and saved in FASTQ format. Quality checks on the fastq files were performed using FastQC and MultiQC. The raw paired-end reads were processed with Trimmomatic to remove low-quality reads, trim adaptor sequences, and filter out poor-quality bases using specific parameters. The resulting high-quality clean reads were used for further analysis. Alignment to the reference genome (H. sapiens, GRCh38) was done using HISAT2 software, and read counts mapped to individual genes were determined with FeatureCounts. For gene expression analysis, normalization procedures such as Trimmed Mean of M-values (TMM) and Relative Log Expression (RLE) were applied using DEGseq and DESeq2, respectively. P-values were adjusted using the Benjamini and Hochberg method to control the false discovery rate (FDR).

### Cut and Tag-Seq

SAS cells (1×10^6^ cells) were seeded in a 10-cm dish and incubated at 37 °C overnight. The cells were collected by trypsinization and then followed the Hyperactive Universal CUT&Tag Assay Kit Illumina (Vazyme, TD904) protocol for experiment and library preparation. The experiment, including quality control, NGS service, and data analysis assistance, were conducted by Topgen Biotechnology Co., Ltd. in Taiwan (R.O.C). The libraries were amplified with 12 cycles on the thermocycler. Post amplification, the libraries were size selected at 250–600 bp in length with DNA Clean Beads (Cat. No. N411, Vazyme Biotech, Nanjing, CN). The amount and length distribution of the library were quantified using microfluidic electrophoresis with DNA-2500 Kit on MultiNA MCE-202 (Shimadzu, Kyoto, JP). The adapter sequences of Cut and Tag-Seq reads were removed, and reads with quality score > 25 and length > 140 were retained using fastq v0.22. Cut and Tag-Seq reads were aligned to the UCSC hg38 genome using Bowtie2 v2.4.5. The mapping of insert length between 10–700 bp is retained as mapped fragments. The mapping of insert length between 10–700 bp is retained as mapped fragments. The fragments were then normalized by chromosome size of the hg38 genome and converted to bedgraph with bedtools v2.30.0. Output bedgraph files were called peaks by SEACR v1.3 with the stringent model of the top 0.01 fraction of peaks based on the total signal within peaks. The called peaks were then annotated by ChIPSeeker v1.28.3. To visualize occupied regions, Galaxy servers (https://usegalaxy.org/) was used further application. ComputeMatrix tools were used for computing the region scoring matrixes, and plotHeatmap tools were used for generating the heatmaps.

### GSEA analysis

The GSEA database (https://www.gsea-msigdb.org/gsea/index.jsp) was used to analyze the correlation between KDM4C and the HALLMARK_HEME_METABOLISM geneset [32]. The SAS-shKDM4C RNA-seq data were analyzed by the GSEA pre-rank function.

### Kaplan-Meier plotter analysis and TCGA database

The overall survival (OS) and relapse-free survival (RFS) of 499 HNSCC cancer patients were analyzed using Kaplan-Meier Plotter online tool (http://kmplot.com/analysis/). Another dataset of HNSCC patients from TCGA PanCancer Atlas (https://datacatalog.mskcc.org/dataset/10415) [33, 34] was also utilized to study the clinical relevance of HNSCC.

### Cytotoxicity assessment via MTT assay

Cells were seeded in 96-well plates (3×10^3^ cells/well) and incubated overnight at 37 °C. Cell viability was measured daily for 4 days using an MTT assay kit (Thermo) and CLARIOstar® Microplate Reader (BMG Labtech, Ortenberg, Germany). Measured optical density at 570 nm.

### Immunoprecipitation (IP)

The cells were collected and broken down using IP lysis buffer (50 mM Tris-HCl (pH 7.4), 150 mM NaCl, 0.5% NP40, and protease inhibitor) at 4 °C for 1.5 hours. The cell lysates, primary antibody (1 μg), and 10 µL PureProteome protein A/G magnetic beads (Millipore) were mixed and incubated at 4 °C overnight with gentle rocking. The beads were washed three times with IP wash buffer (containing 137 mM NaCl, 2.7 mM KCl, 10 mM Na_2_HPO_4_, 1.8 mM KH_2_PO_4_, 0.1% Tween 20, pH 7.4). The protein complexes bound to the beads were then eluted by IP lysis buffer at 85 °C for 10 minutes. The complexes were separated by SDS-PAGE for immunoblotting analysis.

### Chromatin immunoprecipitation (ChIP) assay

The ChIP assay was conducted according to the procedure described in a previous study [35]. The cells were crosslinked using 1% formaldehyde, and the crosslinked chromatin was fragmented to 200‒500 bp by sonication. The lysates were incubated with gentle rocking with Magna ChIP Protein G Magnetic beads (Millipore) and specific antibodies (5 µg) at 4 °C overnight. The ChIP complexes were eluted and analyzed by qRT-PCR. The primer sequences are listed in Table S3. The fold enrichment was calculated using the ΔΔCt method.

### Migration assay in zebrafish

The transgenic zebrafish strain Tg(fli1:EGFP) was obtained from the Zebrafish International Resource Center (ZIRC, Eugene, OR, USA). Embryos, larvae, and adult fish were maintained under standard conditions at the Taiwan Zebrafish Core Facility, NHRI. At 2 days post-fertilization (dpf), embryos were dechorionated and anesthetized with tricaine (0.04 mg/mL). Tumor cells (SAS-LN or FaDu) were labeled with carboxyfluorescein succinimidyl ester (CFSE), and 200 labeled cells (4.6 nL) were injected into the yolk sac using a Nanoject II Auto-Nanoliter Injector (Drummond Scientific, Broomall, PA, USA). Fluorescence imaging was conducted at 1 and 3 days post-injection (dpi) using a Leica fluorescence microscope. At each time point, green fluorescence and bright-field images were captured simultaneously to ensure precise spatial alignment. Image analysis was performed using ImageJ software, where fluorescence and bright-field images were overlaid to localize tumor cells with high accuracy. Tumor cells were considered migratory if they exited the yolk sac, entered the circulation, and localized to distal embryonic regions, specifically the upper neurocoele, notochord, or spinal cord, at 3 dpi. Embryos exhibiting such distal fluorescence signals were classified as having metastatic behavior. Migration was quantified as the percentage of affected embryos across three independent experiments. The total number of embryos analyzed is provided in the Results section. All animal experiments were approved by the Institutional Animal Care and Use Committee (IACUC) of the National Health Research Institutes (NHRI) (Protocol No. IACUC-107057-AC1; Approval Date: 2019/03/08; Trial Period: 2020/01/01 to 2022/12/31). All procedures were conducted in accordance with institutional and national ethical guidelines. The number of animals used was minimized in alignment with the principles of responsible and humane animal research.

### Immunohistochemistry (IHC)

The tissue samples used in this study were 85 cases of consecutive paraffin-embedded human HNSCC tissue microarrays from Taipei Veterans General Hospital (IRB: 2022-01-019 CC). The Immunohistochemistry (IHC) experiments were carried out using NovolinkTM polymer detection system kit (Cat. No. RE7140-K). The biopsies were deparaffinized, rehydrated, and antigen retrieved with 0.01M sodium citrate buffer (pH 6.0). Sample slides were treated with Peroxidase and Protein Block reagents and stained with primary antibodies against KDM4C (NBP1-4900, 1:100) or GATA1 (ab133274, 1:100) overnight. After washing with TBS-T (0.05% Tween 20) three times, they were incubated with Novolink Polymer (anti-rabbit Poly-HRP-IgG) for 30 minutes at room temperature. The slides were then developed with diaminobenzidine and counterstained with Mayer's hematoxylin. Sample slices were scanned using a Pannoramic scanner (Zeiss), and the images were examined by Qupath-0.4.3. The results were reviewed based on two parameters: the staining intensity score (0, negative; 1, weak; 2, moderate; 3, strong) and the percentage of positive-staining cells (0–100). The H score (0–300) was calculated by multiplying these two parameters. Two groups were stratified based on the H score: low expression (H < mean) and high expression (H ≥ mean). Kaplan-Meier analysis was conducted to evaluate the clinical relevance. The correlation between KDM4C and GATA1 was presented as the Pearson R constant.

### Xenograft

SAS cells (1×10^6^ cells) were suspended in PBS (Gibco) and mixed with an equal amount of Matrigel Matrix (Corning, Cat. No. 354234). The mixture was then implanted subcutaneously into a four-week-old female BALB/cAnN.Cg-Foxn1nu/CrlNarl mice (from National Laboratory Animal Center (NAR Labs), Taiwan (R.O.C)). The tumor volume was monitored at regular intervals from one week after implantation. For drug treatment, the tumor volume has waited until it reached 100 mm^3^, and then treatment was started. The tumor volume was calculated using the formula: length (L) × width (W) × height (H) × 0.52. All mice were sacrificed after the experiment ended. The animal studies were approved by the IACUC of National Tsing Hua University (NTHU) (approval number: NTHU-IACUC-110068-1, Approval date: 2022/01/07, Expected trial period: 2022/08/01 to 2025/07/31) and were conducted following institutional guidelines and animal welfare standards. For ethical considerations and animal welfare, we minimized sample sizes to reduce the number of animals used while ensuring statistical significance.

### Docking and simulation

The interaction between KDM4C and GATA1 was analyzed using BIOVIA Discovery Studio 2022 software (BIOVIA, San Diego, CA, USA). To prepare KDM4C (full length, AlphaFold ID: AF-Q9H3R0-F1) and a segment of the GATA1 protein, which includes the N-terminal zinc finger (NZF) and C-terminal zinc finger (CZF) regions (residues 202‒302, AlphaFold ID: AF-P15976-F1), the "Prepare Proteins" tool was used [36, 37]. A minimization procedure was then performed, which updated the molecular coordinates and added energy properties to the proteins. Finally, the "Dock Proteins (ZDOCK)" feature of the software was used to perform protein-protein docking [38, 39]. The presentation of protein structures was applied by PyMol 2.5.2 (Schrödinger, LLC).

### Statistical analysis

For the IHC study, we used the Chi-square test to compare the clinicopathological properties of patients concerning categorical variables. To assess overall patient survival, we employed Kaplan–Meier analysis, which calculated the time from diagnosis until death or last follow-up. The log-rank test was used to determine the statistical significance between groups. Spearman’s correlation was conducted to evaluate the relationship between the status of KDM4C and GATA1 with each clinical parameter. Receiver operating characteristic (ROC) curves were generated based on sensitivity and specificity, and the area under the curve (AUC) was calculated through numerical integration. A two-sided unpaired Student’s t-test was used for two-group comparisons, and ANOVA analysis was used for multiple-group comparisons. The group comparisons and statistical analyses were performed using GraphPad Prism 10 software. A p-value of less than 0.05 was considered statistically significant.

## Results

### High expression of KDM4C correlates with poor prognosis in HNSCC and promotes cancer progression in HNSCC cell models

We utilized the Kaplan-Meier Plotter online tool to investigate the impact of KDM members on the clinical outcomes of HNSCC patients. Our results indicate that high expression of KDM1A (HR: 1.49, 95% CI: 1.09‒2.05, *P* = 0.013), KDM4C (HR: 1.4, 95% CI: 1.06‒1.86, *P* = 0.017), and RIOX2 (HR: 1.61, 95% CI: 1.15‒2.25, *P* = 0.005) is associated with worse overall survival (OS) outcomes (Table S4). Furthermore, elevated expression of KDM4C (HR: 9.82, 95% CI: 1.33‒72.44, *P* = 0.006) and KDM6A (HR: 2.27, 95% CI: 0.99‒5.20, *P* = 0.046) is negatively correlated with relapse-free survival (RFS) outcomes (Table S5). Notably, high expression of KDM4C is significantly linked to worse OS and RFS outcomes, indicating its potential oncogenic role in HNSCC (Fig. S1A, B).

We analyzed the correlation between KDM4C expression and clinical outcomes using The Cancer Genome Atlas (TCGA) data to substantiate these findings further. The analysis confirmed that elevated KDM4C expression was significantly associated with poorer OS and RFS outcomes (Fig. S1C, D). These results provide clinical relevance that KDM4C is overexpressed in HNSCC and its correlation with adverse clinical outcomes.

**Fig. 1:**
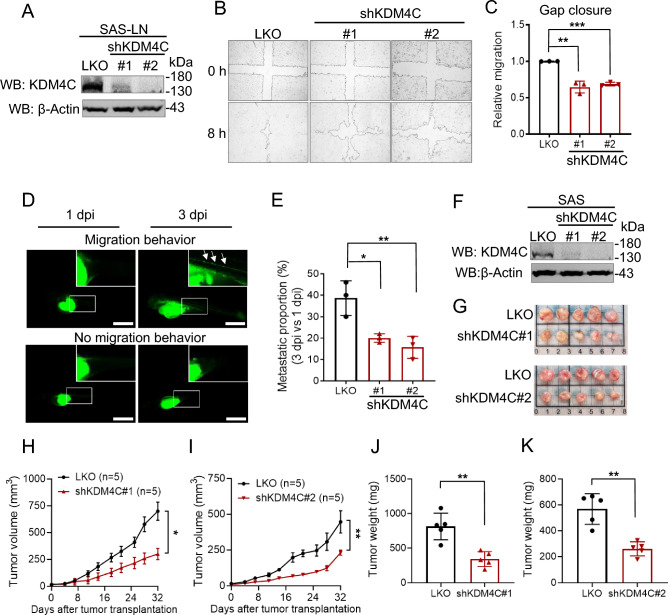
KDM4C promotes cell migration and is crucial for tumor growth in HNSCC. **A** Generation of KDM4C-knockdown (KDM4C-KD) SAS-LN cells. Cells were infected with lentivirus carrying control pLKO.1 (LKO), or shKDM4C constructs (#1 or #2), followed by puromycin selection. KDM4C expression was analyzed by Western blot. Actin was used as the internal control. **B** Wound healing assay of LKO and KDM4C-KD SAS-LN cells. **C** Quantification of the wound closure area from (**B**). **D** Zebrafish xenotransplantation assay of LKO and KDM4C-KD SAS-LN cells. Representative fluorescence images of a zebrafish embryo displaying cell migration (upper, LKO) or no migration (lower, shKDM4C#1) at post-injection (dpi). Scale bar, 1 mm. **E** Quantification of cell migration in zebrafish xenograft from (**D**). Each data point represents the percentage of embryos exhibiting tumor cell migration at 3 dpi in one of three independent biological experiments. The total number of embryos analyzed per group is as follows: LKO (n = 33), shKDM4C#1 (n = 30), shKDM4C#2 (n = 32). **F** Generation of KDM4C-KD SAS cells. KDM4C expression was analyzed by Western blot. Actin was used as the internal control. **G** Examination of LKO and KDM4C-KD SAS cells (1×10^6^ cells) xenografted in BALB/cAnN.Cg-Foxn1nu/Croner mice. **H**, **I** Measurements of tumor volume were recorded twice a week. **J**, **K** Measurements of tumor weight were recorded at the sacrificed endpoint. Data in (**C**, **E**) are represented as individual points and mean, and data in (**H**,** I**) are mean ± SEM. P-values are determined by two-sided Student’s* t*-test (**J**,** K**), one-way ANOVA with Tukey’s multiple comparisons test (**C**,** E**), or two-way ANOVA with Tukey’s multiple comparisons test (**H**,** I**). *P < 0.05, **P < 0.01, ***P < 0.001, *ns* not significant

To explore the biological impact of KDM4C, we employed a lentiviral approach to deplete endogenous KDM4C expression in HNSCC cells. We selected SAS, SAS-LN, and FaDu cells for this study due to their high levels of endogenous KDM4C expression, which aligns with our focus on examining the role of KDM4C in the poor prognosis associated with HNSCC (Fig. S2A). These cell lines are well-established HNSCC models with aggressive phenotypes, such as enhanced wound healing and migration capabilities, making them ideal for studying cancer progression and metastasis. SAS-LN cells were transduced with control (LKO) or knockdown (KD) constructs (shKDM4C#1 and shKDM4C#2). The depletion of KDM4C expression was confirmed in SAS-LN cells (Fig. 1A). In wound healing assays, SAS-LN cells with KDM4C knockdown exhibited significantly slower wound closure than control cells (Fig. 1B, C).

To assess metastatic activity, we utilized an in vivo zebrafish xenotransplantation assay. CFSE-labeled SAS-LN cells (LKO vs. KDM4C-KD) were injected into zebrafish embryos, and their migration was monitored using fluorescence microscopy at 1-day post-injection (1 dpi) and 3 dpi. At 3 dpi, a large proportion of LKO cells had disseminated to distal regions, unlike the KDM4C-KD cells (Fig. 1D). Quantification of embryos with distal fluorescent foci indicated significantly higher metastatic activity in the LKO group compared to the KDM4C-KD groups (Fig. 1E).

We further evaluated whether KDM4C promotes tumor growth using a mouse xenograft model with SAS cells. Mice were subcutaneously injected with either control or KDM4C-KD cells (shKDM4C#1 or #2). Tumor growth was significantly impaired in the KDM4C-KD group compared to controls (Fig. 1F‒K).

These findings were corroborated using FaDu cells. The depletion of KDM4C (shKDM4C#1 or #2) significantly reduced their wound-healing capacity (Fig. S2A‒C), metastatic activity in the zebrafish xenotransplantation assay (Fig. S2D, E), and tumor growth in the mouse xenograft model (Fig. S2F, G). These results collectively suggest that KDM4C promotes metastatic activity and tumor growth in HNSCC.

### KDM4C and GATA1 as regulators of heme-metabolism genes in HNSCC

**Fig. 2:**
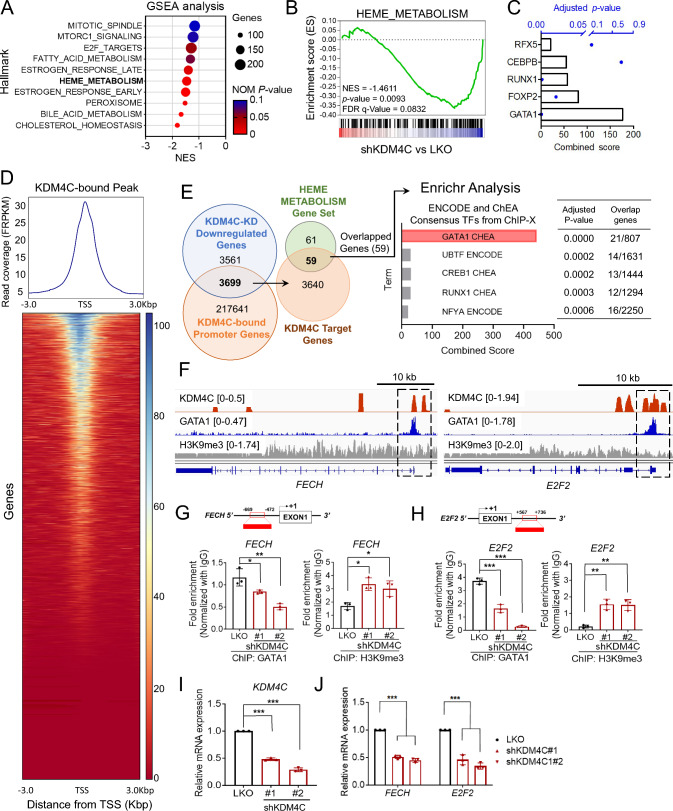
Heme-metabolism genes are regulated by KDM4C and GATA1. **A** Top six KDM4C-correlated pathways from GSEA analysis of KDM4C knockdown (KD) RNA-seq result. **B** KDM4C-KD SAS cells enrich for gene signatures characteristic of heme-metabolism loss, as identified by mRNA-seq followed by GSEA. **C** Enrichr analysis of key transcription factors in heme-metabolism genes derived from (**A**). **D** Global binding profile of KDM4C at transcription start sites (TSSs) within ±3 kb regions, visualized as a heatmap and peak density plot derived from KDM4C CUT&Tag-seq analysis. The heatmap highlights the binding intensity of KDM4C across TSSs, with color gradients representing varying binding levels. Data visualization was generated using the Galaxy plot heatmap tool, allowing for a clear illustration of KDM4C enrichment at promoter regions, indicative of its regulatory role in gene expression. **E** Identification of heme metabolism genes regulated by KDM4C. The Venn diagram depicts the overlap between KDM4C-downregulated genes and KDM4C-bound promoter genes. The intersecting set represents KDM4C-regulated genes involved in heme metabolism. An Enrichr analysis (https://maayanlab.cloud/Enrichr/) was performed on these genes to identify consensus transcription factors, with GATA1 emerging as the top-ranking transcription factor, highlighted in the enrichment bar chart to indicate its pivotal role in the KDM4C regulatory network. **F** KDM4C (this study), GATA1 (SRX4172742_HCT116), and H3K9me3 (ENCFF794 WNF) at proximal promoter regions of *FECH* and *E2F2*.** G**,** H** Analysis of occupancy changes at the promoter region of *FECH* (**G**) and *E2F2* (**H**) via ChIP-qPCR. ChIP was conducted with specific antibodies (anti-GATA1 and anti-H3K9me3) in LKO and KDM4C-KD SAS cells. **I, J** The relative mRNA levels of heme-metabolism genes (*FECH* and *E2F2*) in the LKO and KDM4C-KD SAS cells. Data in (**G**–**J**) are represented in individual points and mean. P-values are determined by one-way ANOVA with Tukey’s multiple comparisons test (**G**–**I**), or two-way ANOVA with Tukey’s multiple comparisons test (**J**). *P < 0.05, **P < 0.01, ***P < 0.001, *ns* not significant

To understand the epigenetic regulation of KDM4C, we conducted RNA-seq analysis comparing KDM4C knockdown (shKDM4C) and control (LKO) SAS cells. To systematically assess the biological pathways influenced by KDM4C depletion, we conducted GSEA analysis, which revealed significant downregulation of multiple oncogenic pathways as shown in Fig. 2A. Among these, heme metabolism emerged as a significantly downregulated pathway, an interesting finding given that the impact of KDM4C on heme metabolism remains largely unexplored.

Heme metabolism plays a critical role in cellular bioenergetics, oxidative stress regulation, and tumor progression, making it highly relevant in cancer biology. Our GSEA results revealed a negative normalized enrichment score (NES = −1.4611, *P* = 0.0093), indicating a significant reduction in the expression of heme metabolism-related genes upon KDM4C knockdown (Fig. 2B). This finding prompted us to investigate the potential transcriptional regulators involved in this process. Since KDM4C is an epigenetic regulator, we hypothesized that it cooperates with a transcription factor to modulate heme metabolism gene expression. To identify candidate transcription factors, we performed enrichment analysis using Enrichr, which pinpointed GATA1 as the top-ranked transcription factor associated with the heme metabolism gene set (Fig. 2C). GATA1 is a well-known regulator of erythropoiesis and heme biosynthesis, suggesting a functional interaction between KDM4C and GATA1 in heme metabolism regulation in HNSCC.

We performed KDM4C-CUT&Tag seq analysis on SAS cells to investigate the genome-wide distribution of KDM4C. Our results indicated that KDM4C binding was predominantly concentrated around transcription start sites (TSS) (Fig. 2D). Using a Venn diagram, we analyzed the overlap between KDM4C-CUT&Tag seq promoter genes and downregulated genes from the RNA-seq data, revealing 3699 common genes (Fig. 2E). Of these, 59 genes were related to heme metabolism according to the GSEA dataset. Further analysis with Enrichr confirmed GATA1 as the top transcription factor, with a combined score of 443.5. Among the identified genes, 21 were associated with heme metabolism, including *E2F2*, a key player in cell cycle progression and DNA replication and repair, and *FECH*, the terminal enzyme in the heme biosynthesis pathway responsible for catalyzing the insertion of iron into protoporphyrin IX to form heme [40]. These findings suggest that KDM4C and GATA1 orchestrate a regulatory network modulating heme metabolism genes in HNSCC.

Using combined KDM4C (this study) and GATA1 (SRX4172742_HCT116) ChIP data, we observed overlapping signals at the proximal promoter regions of heme-metabolism genes *FECH* and *E2F2*. This occupancy was accompanied by reduced levels of repressive H3K9me3 mark (ENCFF794 WNF) at these loci (Fig. 2F). These results suggest that KDM4C and GATA1 are co-recruited to the promoters of heme-metabolism genes and work together to regulate heme metabolism.

To functionally validate the KDM4C-GATA1 interaction, we performed ChIP-qPCR analysis of SAS cells with KDM4C knockdown (shKDM4C#1 or #2). Depletion of KDM4C significantly reduced GATA1 occupancy at the *FECH* promoter and increased the deposition of the repressive H3K9me3 mark (Fig. 2G). A similar pattern was observed at the *E2F2* locus (Fig. 2H). qRT-PCR analysis further confirmed reduced expression of *FECH* and *E2F2* upon KDM4C knockdown (Fig. 2I, J).

These results were further corroborated in FaDu cells, where KDM4C knockdown significantly reduced GATA1 recruitment and enhanced H3K9me3 signals at the promoter regions of *FECH* and *E2F2* (Fig. S3A, B). This depletion also led to decreased expression of *FECH* and *E2F2* (Fig. S3C, D).

These results suggest that KDM4C and GATA1 work in concert to regulate heme metabolism gene transcription. We propose that KDM4C may serve as a coactivator of GATA1, facilitating an open chromatin structure to recruit GATA1 to the promoter region. Given the essential role of heme metabolism in mitochondrial function, its upregulation may contribute to wound healing activity by supplying prosthetic groups for oxidative phosphorylation complexes in mitochondrial respiration [19–21]. Recent evidence has also suggested that GATA1 and heme work together to sustain high mitochondrial bioenergetics, essential for the rapid growth and spread of cancer cells [22, 41]. Our findings support the upregulation of heme metabolism through the GATA1-KDM4C pathway as a key factor in promoting HNSCC metastasis.

To further substantiate our findings and expand the context of KDM4C/GATA1's regulatory role in HNSCC, we analyzed the relationship between heme metabolism and other cancer-related pathways using the TCGA HNSCC dataset. We calculated Heme Metabolic, Proliferation, and Metastasis scores through single-sample Gene Set Enrichment Analysis (ssGSEA) and conducted Pearson correlation analyses. Our results demonstrate a positive correlation between Heme Metabolic scores and Proliferation and Metastasis scores (Fig. S4A, B), suggesting that increased heme metabolism is associated with enhanced proliferative and metastatic potential in HNSCC.

Additionally, we assessed the clinical significance of GATA1 expression in HNSCC patients using the TCGA dataset. Kaplan-Meier survival analysis demonstrated that patients with high GATA1 expression exhibited significantly worse relapse-free survival compared to those with low GATA1 expression (Fig. S4C, D), reinforcing the role of GATA1 in tumor progression. Furthermore, patients with co-upregulation of KDM4C and GATA1 had significantly poorer overall survival compared to those with lower expression of both genes (Fig. S4E, F). These findings suggest that the interplay between KDM4C and GATA1 contributes to HNSCC malignancy by regulating heme metabolism and its associated oncogenic pathways [17].

### Interaction between KDM4C and GATA1

**Fig. 3:**
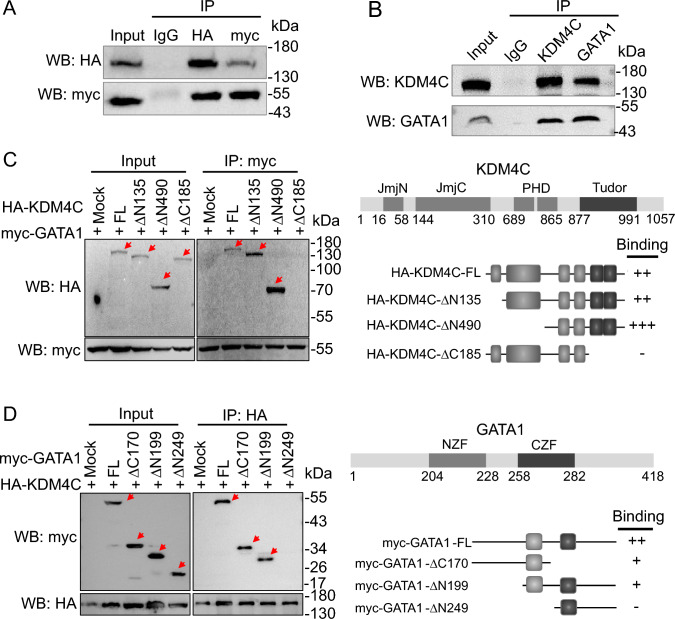
The interaction of KDM4C and GATA1. **A** Association of ectopically expressed KDM4C and GATA1. Western blot analysis of HEK293T cells co-transfected with pcDNA3.1-HA-KDM4C and pcDNA3.1-myc-his-GATA1 as indicated. Immunoprecipitation (IP) was conducted using anti-HA or anti-myc antibody. Rabbit/mouse IgG (1:1) was used as a negative control. **B** Western blot analysis of endogenous IP in SAS cells. IP was conducted using anti-KDM4C or anti-GATA1 antibody. Rabbit IgG was used as negative control. **C** Co-IP assays were performed with an anti-myc antibody in lysates from HEK293T cells co-transfected with a full-length (FL) myc-GATA1 vector plus one of the HA-tagged constructs [mock, full-length (FL) KDM4C, or KDM4C truncated mutants (∆N135, ∆N490 and ∆C185)], followed by western blot analysis. **D** Co-IP assays were performed with an anti-HA antibody in lysates from HEK293T cells co-transfected with a full-length (FL) HA-KDM4C vector plus one of the myc-tagged constructs [mock, full-length (FL) GATA1, or GATA1 truncated mutants (∆C170, ∆N199 and ∆N249)], followed by western blot analysis

We investigated the possibility that KDM4C and GATA1 form a complex, as coactivators often associate directly with transcription factors. Immunoprecipitation (IP) analysis using lysates from HEK293T cells co-transfected with pcDNA3.1-HA-KDM4C and pcDNA3.1-myc-GATA1 showed a clear interaction between KDM4C and GATA1 (Fig. 3A). The interaction between KDM4C and GATA1 was further substantiated through endogenous immunoprecipitation assays conducted in SAS (Fig. 3B) and FaDu (Fig. S5A). These results suggest the KDM4C-GATA1 interaction within a more pertinent cellular context, enhancing the relevance of the findings.

We used full-length myc-tagged GATA1 and various HA-tagged KDM4C constructs (full-length, ∆N135, ∆N490, and ∆C185) to determine the specific regions responsible for this interaction. IP assays with an anti-myc antibody on these lysates revealed that the ∆C185 KDM4C mutant lost its binding ability, indicating that the C-terminal region of KDM4C is crucial for the interaction (Fig. 3C). Further IP assays using myc-tagged GATA1 variants (full-length, ∆C170, ∆N199, and ∆N249) and full-length HA-tagged KDM4C showed that the ∆N249 GATA1 mutant exhibited no binding signal (Fig. 3D).

An additional IP experiment was conducted using HA-tagged KDM4C-∆N490 and different myc-tagged GATA1 constructs (full-length GATA1, GATA1 ∆C170, and GATA1 ∆N249) to map the interaction region between KDM4C and GATA1. The results demonstrated that KDM4C-∆N490 interacts with full-length GATA1 and GATA1 ∆C170 but not with GATA1 ∆N249, confirming that the critical binding site of GATA1 resides within its N-terminal domain (Fig. S5B). Docking analysis further supported these findings by illustrating that the interaction between the Tudor domain of KDM4C and the NZF domain of GATA1 is mediated primarily through hydrogen bonds and electrostatic interactions, providing a structural basis for this interaction (Fig. S5C).

To assess the binding affinity between KDM4C and GATA1, we attempted to express and purify the recombinant KDM4C-∆N490 and GATA1-∆C170 proteins in the *E. coli* system. However, due to limitations in protein yield and purity in our system (Fig. S5D), we utilized a biochemical binding assay to qualitatively and semi-quantitatively evaluate the interaction between KDM4C and GATA1. This assay confirmed the specific binding between the two proteins (Fig. S5E). Although this approach does not yield precise binding affinity values, we estimated the protein concentration at which 50% binding occurred as an approximate measure of interaction strength.

These findings collectively indicate that KDM4C interacts directly with GATA1 through the Tudor domain of KDM4C and the NZF region of GATA1, supporting the functional significance of this interaction in heme metabolism regulation.

### The KDM4C/GATA1-FECH axis promotes HNSCC progression

To further investigate the role of heme metabolism in HNSCC, we selected ferrochelatase encoded by *FECH*, an essential enzyme in the heme biosynthesis pathway, as a representative gene. *FECH* depletion (shFECH#1 or #2) was performed in SAS and FaDu cell lines (Fig. S6A, B). The results demonstrated that *FECH* depletion significantly impaired cell migration in wound healing assays (Fig. S6C–F), invasion assays (Fig. S6G, H), and proliferation assays (Fig. S6I, J). These findings suggest that *FECH* plays a crucial role in HNSCC.

**Fig. 4:**
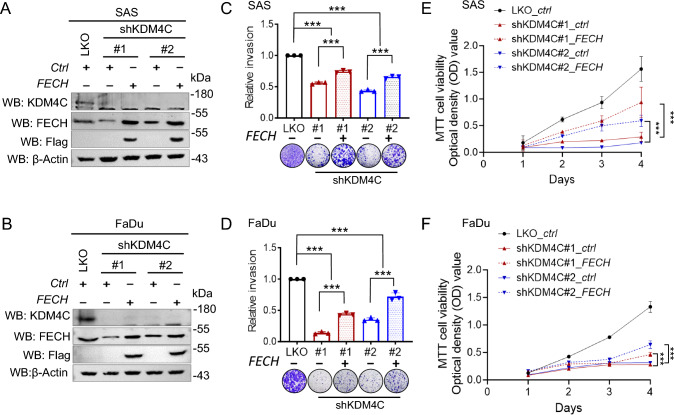
Effect of FECH overexpression on invasion and proliferation in KDM4C-knockdown SAS and FaDu cells. **A, B** Analysis of FECH expression in LKO and KDM4C-knockdown (shKDM4C#1 or #2) SAS (**A**) and FaDu (**B**) cells. Cells were transfected with control or Flag-FECH expression vector, followed by Western blot analysis. **C**,** D** Invasion assay of LKO and FECH-overexpressing KDM4C-KD SAS (**C**) and FaDu (**D**) cells. **E**, **F** MTT cell proliferation assay of LKO and FECH-overexpressing KDM4C-KD SAS (**E**) and FaDu (**F**) cells at indicated time points. Data in (**C**,** D**) are represented in individual points and mean, and data in (**E**,** F**) are mean ± SD. P-values are determined by one-way ANOVA with Tukey’s multiple comparisons test (**C**, **D**) and two-way ANOVA with Tukey’s multiple comparisons test (**E**,** F**). *P < 0.05, **P < 0.01, ***P < 0.001, *ns* not significant

Next, we investigated the hypothesis that the KDM4C/GATA1-FECH axis contributes to HNSCC progression. We overexpressed *FECH* using a Flag-tag construct in KDM4C knockdown SAS and FaDu cells. The depletion of KDM4C impaired cell invasion and proliferation, while overexpression of *FECH* in KDM4C-depleted SAS or FaDu cells restored these abilities (Fig. 4A‒F). Similarly, invasion and proliferation assays were conducted on GATA1-depleted cells. A reduction in invasion and proliferation was observed in GATA1 knockdown SAS and FaDu cells, but overexpression of *FECH* rescued these phenotypes (Fig. S7A‒F).

We further conducted experiments using siRNA-mediated knockdown of KDM4C and GATA1 (siKDM4C and siGATA1) to validate the previous findings. The gene expression levels of *E2F2* and *FECH*, as well as migration assay results, were consistent with those observed in the shRNA experiments (Fig. S8A‒D, Fig. S9A‒D). Additionally, rescuing *FECH* expression in cells with siKDM4C or siGATA1 knockdown restored their proliferation and invasion capabilities (Fig. S8E‒G, Fig. S9E‒G). These findings strengthen our hypothesis regarding the critical role of the KDM4C/GATA1-FECH regulatory axis in HNSCC progression.

### High KDM4C expression in HNSCC correlates with poor prognosis and GATA1 co-expression

**Fig. 5:**
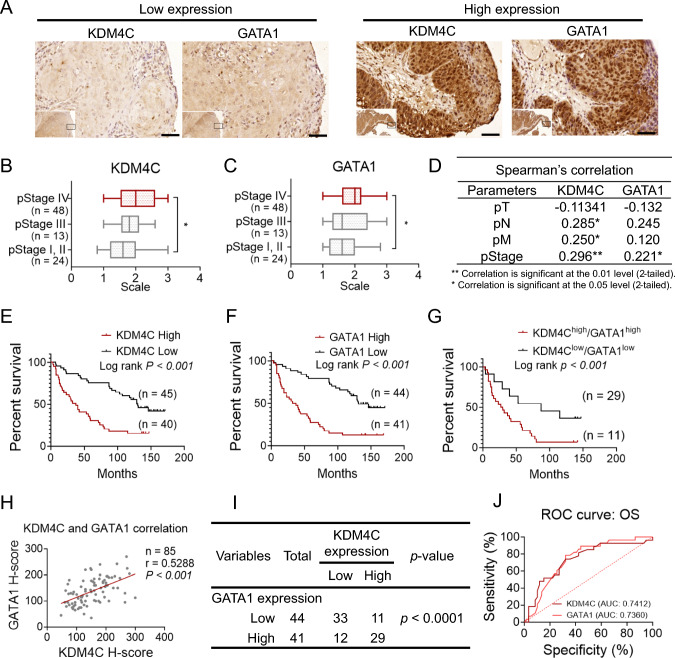
Correlation and clinical impact of KDM4C and GATA1 expression in HNSCC. **A** Representative immunohistochemical staining for KDM4C and GATA1 in tumor slides from HNSCC patients. Scale bar = 50 μm. **B, C** Distribution of KDM4C and GATA1 staining intensity across different pStage groups. **D** Spearman’s correlation analysis of KDM4C and GATA1 expression with clinical stages in HNSCC (pT: primary tumor extent, pN: regional lymph node metastasis, pM: distant metastasis). **E**‒**G** Kaplan-Meier survival curves demonstrating the association of high expression of KDM4C (**E**), GATA1 (**F**), and both (**G**) with reduced overall survival in HNSCC patients. Grouping is based on H-score averages for high/low expression. **H** Pearson correlation analysis between KDM4C expression and GATA1 levels in 85 HNSCC cases. **I** Chi-square test assessing the statistical significance of the correlation between KDM4C and GATA1. **J** Receiver operating characteristic (ROC) curve analysis evaluating the diagnostic potential of KDM4C and GATA1 in HNSCC. P-values are determined by a two-tailed Student’s t-test for group comparisons (**A**, **B**). *P < 0.05, **P < 0.01, ***P < 0.001, *ns* not significant

To further investigate the clinical relevance of KDM4C and GATA1, we conducted an immunohistochemical (IHC) analysis on 85 paraffin-embedded oral cancer specimens from Taipei Veterans General Hospital patients. Representative staining images of high and low expression groups for KDM4C and GATA1 are shown in Fig. 5A. Expression levels were quantified using the IHC H-score, calculated by multiplying intensity by proportion. Our results indicated that KDM4C expression is significantly higher in patients with advanced-stage (pStage IV) compared to those in early stages (Fig. 5B). A similar trend was observed for GATA1 expression (Fig. 5C). Spearman’s coefficient analysis revealed a significant correlation between KDM4C expression and clinical parameters (pN, pM, and pStage) (Fig. 5D), suggesting a strong association between KDM4C and cancer progression.

Additionally, Kaplan-Meier survival analysis showed that patients with high KDM4C expression had significantly poorer outcomes than those with low expression (Fig. 5E). A similar pattern was observed for GATA1 expression (Fig. 5F). Moreover, patients with high co-expression levels of both KDM4C and GATA1 had worse outcomes than those with low expression of both markers (Fig. 5G). A significant correlation was found between KDM4C and GATA1 expression levels (Pearson r = 0.5288, Fig. 5H), which was further supported by Chi-square test results (Fig. 5I). Receiver Operating Characteristic (ROC) analysis demonstrated that KDM4C (AUC = 0.7412) and GATA1 (AUC = 0.7360) are effective diagnostic markers in HNSCC (Fig. 5J).

### KDM4 inhibition by myricetin and its analogue 22S0 suppresses HNSCC progression

**Fig. 6:**
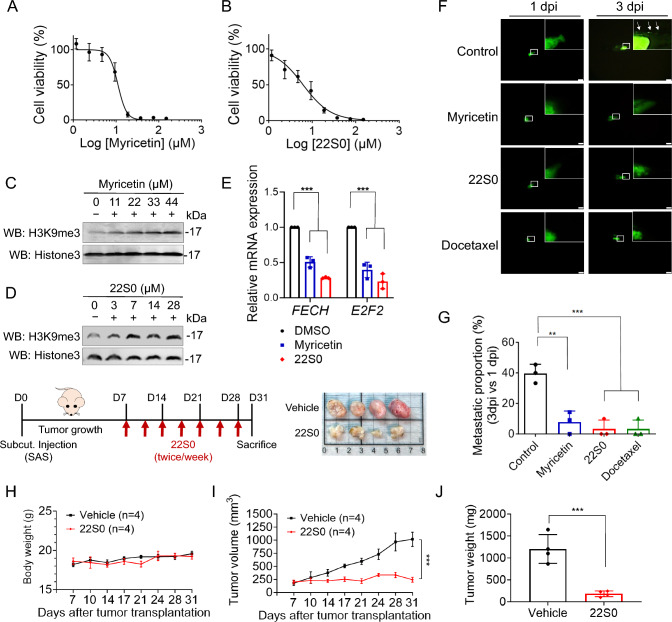
Effects of KDM4 inhibitors on heme metabolism and tumor growth in HNSCC. **A**, **B** SAS-LN cells were treated with varying concentrations of myricetin (**A**) or 22S0 (**B**) for 3 days. Cell survival rates were measured using the MTT assay and are shown as dose-response curves. **C**, **D** Analysis of H3K9me3 levels in inhibitor-treated SAS-LN cells for 3 days. The H3K9me3 signals were detected by Western blot analysis. **E** Relative mRNA levels of heme metabolism genes (*FECH* and *E2F2*) in SAS-LN cells following 24-hour treatment with control (DMSO, 0.1%), myricetin (12.5 µM), or 22S0 (6.25 µM). **F** Representative images of SAS-LN cells xenografted in zebrafish and treated with drugs. Treatments include control (DMSO, 0.1%), myricetin (11.07 µM), 22S0 (6.17 µM), and docetaxel (0.38 nM). Scale bar: 200 µm. **G** Quantification of cell migration of SAS-LN cells under various drug treatments from (**F**). Each data point represents the percentage of embryos exhibiting tumor cell migration at 3 dpi in one of three independent biological experiments. The total number of embryos analyzed per group is as follows: control (n = 33), myricetin (n = 41), 22S0 (n = 32), and docetaxel (n = 31). **H**‒**J** SAS cells (1×10^6^ cells) were subcutaneously injected into the BALB/cAnN.Cg-Foxn1nu/CrlNarl mice. When the tumors had grown to approximately 100 mm^3^, the mice received treatment. They were given two injections per week, with either 75 mg/kg of 22S0 or a control vehicle solution (DMSO/PEG300/PBS), administered via intra-tumor injection. Tumor volumes (**H**) and body weights (**I**) were measured at each treatment session, and tumor weights were measured at sacrificed endpoint (**J**). Data in (**E**, **G**, **J**) are represented as individual points and mean. Data in (**A**, **B**) are represented in mean ± SD, and data in (**H**, **I**) are mean ± SEM. P-values are determined by two-way ANOVA with Tukey’s multiple comparisons test (**E**,** I**), one-way ANOVA with Tukey’s multiple comparisons test (**G**), and two-tailed Student’s t–test (**J**). *P < 0.05, **P < 0.01, ***P < 0.001, *ns* not significant

We have previously demonstrated that myricetin, a natural compound, effectively inhibits KDM4 activity by binding to its catalytic site [42]. To further explore the potential of myricetin and its analogue BPRKD022S0 (22S0) in treating HNSCC, we conducted cytotoxicity assays [42]. Both myricetin and 22S0 exhibited cytotoxicity against SAS-LN cells (myricetin, IC_50_ = 11.07 ± 0.55 μM; 22S0, IC_50_ = 6.17 ± 0.35 μM) (Fig. 6A, B). A similar trend was observed in FaDu cells (myricetin, IC_50_ = 13.37 ± 0.92 μM; 22S0, IC_50_ = 4.11 ± 0.34 μM) (Fig. S10A, B).

We observed that treatment with KDM4 inhibitors led to an increase in H3K9me3 levels in SAS-LN (Fig. 6C, D) and FaDu (Fig. S10C, D) cells, indicating effective inhibition of KDM4 demethylase activity. Further investigation revealed that both myricetin and 22S0 downregulated the mRNA levels of heme metabolism genes (*FECH* and *E2F2*) in SAS-LN (Fig. 6E) and FaDu (Fig. S10E) cells, suggesting that KDM4 inhibition reduces heme metabolism gene expression.

We used a zebrafish xenotransplantation assay to assess the effects on metastatic activity. Treatment with myricetin, 22S0, and docetaxel significantly reduced cell dissemination (vehicle: 39.6%; myricetin: 7.79%; 22S0: 3.33%; docetaxel: 3.33%) in SAS-LN-injected embryos (Fig. 6F, G). Similar results were observed in FaDu-injected embryos (Fig. S10F, G). In a SAS xenograft model, 22S0 treatment significantly impaired tumor growth compared to the vehicle group (Fig. 6H‒J). These findings demonstrate that myricetin and 22S0 effectively inhibit KDM4 activity, reducing cell metastasis and tumor growth.

To evaluate the specificity of KDM4C in regulating heme metabolism genes, we performed qRT-PCR analysis on cells depleted of KDM4A (shKDM4A#1 or #2) and KDM4B (shKDM4B#1 or #2) compared to KDM4C-KD cells. The expression levels of heme metabolism genes (*E2F2* and *EFCH*) were significantly reduced in KDM4C-KD cells than in KDM4A-KD or KDM4B-KD cells (Fig. S11). This indicates that the downregulation of heme metabolism genes is primarily mediated by KDM4C dysfunction, highlighting the specific role of KDM4C in regulating these genes.

## Discussion

**Fig. 7:**
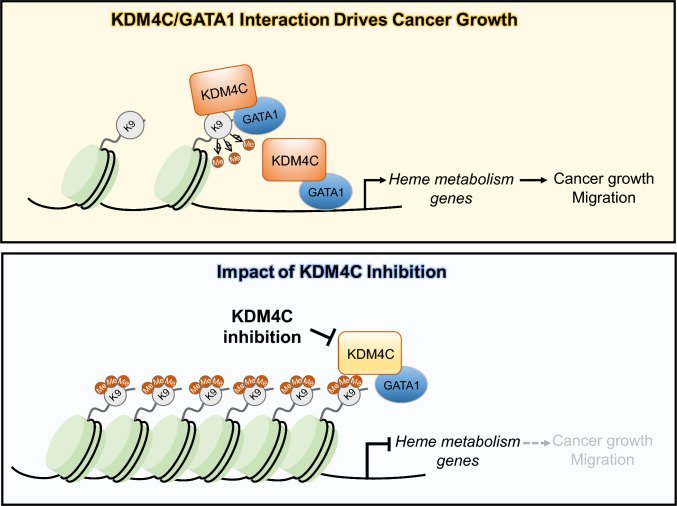
Schematic illustration of KDM4C-GATA1 axis in heme metabolism and cancer progression. In the active state (top panel), KDM4C interacts with GATA1, demethylating H3K9me3 marks at heme metabolism gene promoters such as *FECH*, opening chromatin, and promoting gene transcription. This upregulation supports cancer cell growth and migration. Upon KDM4C inhibition (bottom panel), H3K9me3 demethylation is blocked, leading to a repressive chromatin state, downregulating heme metabolism genes, and reducing cancer growth and migration

Strategies for combating resistance mediated by non-genetic adaptation remain limited compared to targeting drug-resistant mutant cancer cells [43, 44]. Our findings suggest the KDM4C-GATA1 axis is a key regulator of heme metabolism, which promotes tumor growth and metastasis in HNSCC. Mechanistically, KDM4C recruits GATA1 to heme metabolism genes and facilitates transcriptional activation through H3K9me3 demethylation. This epigenetic adaptability may enable cancer cells to reprogram metabolic pathways, promoting survival and progression (Fig. 7).

Analysis of an HNSCC patient cohort from TVGH and TCGA datasets revealed a strong association between KDM4C/GATA1 co-expression and poor overall survival. Moreover, tumor cells with a ferroptosis signature, influenced by heme metabolism, may modulate immune responses, potentially enhancing the effectiveness of immune checkpoint inhibitors [45]. These findings suggest an unexplored link between KDM4C, GATA1, heme metabolism, and cancer progression.

Our data indicate that KDM4C and GATA1 cooperate to upregulate FECH, the terminal enzyme in heme biosynthesis [46], enhancing mitochondrial bioenergetics, oxidative phosphorylation, and supporting tumor aggression [20, 41]. Notably, FECH knockdown significantly impaired migration, invasion, and proliferation, reinforcing the importance of the KDM4C/GATA1-FECH axis.

Our study further deciphers the structural basis of this interaction. KDM4C's Tudor domain binds to GATA1's NZF, a region also involved in interactions with FOG1 and LMO2, suggesting that GATA1’s NZF may serve as a regulatory core for transcriptional complexes [47, 48]. Structural modeling and binding analysis indicate that this interaction is stabilized by hydrogen bonds and electrostatic interactions, providing potential avenues for small-molecule disruption.

Given the oncogenic role of KDM4C in various cancers [24, 35, 49–51], we evaluated its therapeutic potential using small-molecule inhibitors. Myricetin and its analogue 22S0 effectively inhibit KDM4C activity [28, 49], with 22S0 demonstrating promising anti-tumor efficacy in zebrafish and mouse xenograft models. These findings reinforce the potential of KDM4C inhibitors as therapeutic agents for HNSCC [52, 53].

Beyond HNSCC, GATA1 has been implicated in breast and bladder cancers [29], suggesting its role may extend to other solid tumors. Further research should explore whether KDM4C-GATA1 interactions regulate similar oncogenic pathways across different cancer types. Additionally, combining KDM4 inhibitors with immune checkpoint blockade may enhance treatment efficacy by modulating the tumor microenvironment.

Our findings suggest the KDM4C/GATA1 interaction might function as a broader integrative hub beyond the heme metabolism, potentially interacting with other epigenetic regulators and transcription factors involved in oxidative stress and iron metabolism [54]. These alternative pathways warrant further investigation to develop combinatorial therapeutic strategies.

In conclusion, this study provides a deeper understanding of the KDM4C/GATA1-FECH axis in HNSCC, linking epigenetic regulation and metabolic reprogramming. Our results support the therapeutic potential of KDM4C inhibition and provide a rationale for targeting heme metabolism in aggressive cancers. Future studies should explore broader oncogenic interactions and validate KDM4C-targeted therapies in clinical settings.

## Supplementary Information

Below is the link to the electronic supplementary material.

Supplementary file1 (DOCX 3746 KB)

## Data Availability

The data generated in this study are available in GEO at GSE252493 and GSE273500. The ChIP-seq data analysis of GATA1 in HCT116 in this study was obtained from ChIP-Atlas (https://chip-atlas.org/view?id=SRX4172742) and H3K9me3 was obtained from ENCODE (https://www.encodeproject.org/experiments/ENCSR101KMF/).

## References

[1] Johnson DE, Burtness B, Leemans CR et al (2020) Head and neck squamous cell carcinoma. Nat Rev Dis Primers 6:92. 10.1038/s41572-020-00224-3 33243986 10.1038/s41572-020-00224-3 PMC7944998

[2] Garavello W, Ciardo A, Spreafico R et al (2006) Risk factors for distant metastases in head and neck squamous cell carcinoma. Arch Otolaryngol Head Neck Surg 132:762–766. 10.1001/archotol.132.7.762 16847186 10.1001/archotol.132.7.762

[3] Bhatia A, Burtness B (2023) Treating head and neck cancer in the age of immunotherapy: a 2023 update. Drugs 83:217–248. 10.1007/s40265-023-01835-2 36645621 10.1007/s40265-023-01835-2

[4] Wang H, Zheng Z, Zhang Y et al (2023) Locally advanced head and neck squamous cell carcinoma treatment efficacy and safety: a systematic review and network meta-analysis. Front Pharmacol 14:1269863. 10.3389/fphar.2023.1269863 37795033 10.3389/fphar.2023.1269863 PMC10546034

[5] Hanahan D, Weinberg RA (2000) The hallmarks of cancer. Cell 100:57–70. 10.1016/s0092-8674(00)81683-9 10647931 10.1016/s0092-8674(00)81683-9

[6] Hanahan D (2022) Hallmarks of cancer: new dimensions. Cancer Discov 12:31–46. 10.1158/2159-8290.CD-21-1059 35022204 10.1158/2159-8290.CD-21-1059

[7] Cheng Y, He C, Wang M et al (2019) Targeting epigenetic regulators for cancer therapy: mechanisms and advances in clinical trials. Signal Transduct Target Ther 4:62. 10.1038/s41392-019-0095-0 31871779 10.1038/s41392-019-0095-0 PMC6915746

[8] Shi J, Xu J, Chen YE et al (2021) The concurrence of DNA methylation and demethylation is associated with transcription regulation. Nat Commun 12:5285. 10.1038/s41467-021-25521-7 34489442 10.1038/s41467-021-25521-7 PMC8421433

[9] Greer EL, Shi Y (2012) Histone methylation: a dynamic mark in health, disease and inheritance. Nat Rev Genet 13:343–357. 10.1038/nrg3173 22473383 10.1038/nrg3173 PMC4073795

[10] Kooistra SM, Helin K (2012) Molecular mechanisms and potential functions of histone demethylases. Nat Rev Mol Cell Biol 13:297–311. 10.1038/nrm3327 22473470 10.1038/nrm3327

[11] Labbé RM, Holowatyj A, Yang ZQ (2014) Histone lysine demethylase (KDM) subfamily 4: structures, functions and therapeutic potential. Am J Transl Res 6:1–15 PMC3853420 24349617

[12] Morera L, Lubbert M, Jung M (2016) Targeting histone methyltransferases and demethylases in clinical trials for cancer therapy. Clin Epigenetics 8:57. 10.1186/s13148-016-0223-4 27222667 10.1186/s13148-016-0223-4 PMC4877953

[13] Diao WF, Zheng JB, Li Y et al (2022) Targeting histone demethylases as a potential cancer therapy. Int J Oncol 61:103. 10.3892/ijo.2022.5393 35801593 10.3892/ijo.2022.5393

[14] Srivastava R, Singh R, Jauhari S et al (2023) Histone demethylase modulation: epigenetic strategy to combat cancer progression. Epigenomes 7:10. 10.3390/epigenomes7020010 37218871 10.3390/epigenomes7020010 PMC10204559

[15] DeBerardinis RJ, Chandel NS (2016) Fundamentals of cancer metabolism. Sci Adv 2:e1600200. 10.1126/sciadv.1600200 27386546 10.1126/sciadv.1600200 PMC4928883

[16] Pavlova NN, Zhu J, Thompson CB (2022) The hallmarks of cancer metabolism: still emerging. Cell Metab 34:355–377. 10.1016/j.cmet.2022.01.007 35123658 10.1016/j.cmet.2022.01.007 PMC8891094

[17] Fiorito V, Chiabrando D, Petrillo S et al (2019) The multifaceted role of heme in cancer. Front Oncol 9:1540. 10.3389/fonc.2019.01540 32010627 10.3389/fonc.2019.01540 PMC6974621

[18] Swenson SA, Moore CM, Marcero JR et al (2020) From synthesis to utilization: the ins and outs of mitochondrial heme. Cells 9:579. 10.3390/cells9030579 32121449 10.3390/cells9030579 PMC7140478

[19] Sohoni S, Ghosh P, Wang TY et al (2019) Elevated heme synthesis and uptake underpin intensified oxidative metabolism and tumorigenic functions in non-small cell lung cancer cells. Cancer Res 79:2511–2525. 10.1158/0008-5472.Can-18-2156 30902795 10.1158/0008-5472.CAN-18-2156

[20] Kalainayakan SP, FitzGerald KE, Konduri PC et al (2018) Essential roles of mitochondrial and heme function in lung cancer bioenergetics and tumorigenesis. Cell Biosci 8:56. 10.1186/s13578-018-0257-8 30410721 10.1186/s13578-018-0257-8 PMC6215344

[21] Hooda J, Cadinu D, Alam MM et al (2013) Enhanced heme function and mitochondrial respiration promote the progression of lung cancer cells. PLoS One 8:e63402. 10.1371/journal.pone.0063402 23704904 10.1371/journal.pone.0063402 PMC3660535

[22] Sica V, Bravo-San Pedro JM, Stoll G et al (2020) Oxidative phosphorylation as a potential therapeutic target for cancer therapy. Int J Cancer 146:10–17. 10.1002/ijc.32616 31396957 10.1002/ijc.32616

[23] Boreel DF, Span PN, Heskamp S et al (2021) Targeting oxidative phosphorylation to increase the efficacy of radio- and immune-combination therapy. Clin Cancer Res 27:2970–2978. 10.1158/1078-0432.CCR-20-3913 33419779 10.1158/1078-0432.CCR-20-3913

[24] Berry WL, Janknecht R (2013) KDM4/JMJD2 histone demethylases: epigenetic regulators in cancer cells. Cancer Res 73:2936–2942. 10.1158/0008-5472.Can-12-4300 23644528 10.1158/0008-5472.CAN-12-4300 PMC3655154

[25] Lee DH, Kim GW, Jeon YH et al (2020) Advances in histone demethylase KDM4 as cancer therapeutic targets. FASEB J 34:3461–3484. 10.1096/fj.201902584R 31961018 10.1096/fj.201902584R

[26] Ferreira R, Ohneda K, Yamamoto M et al (2005) GATA1 function, a paradigm for transcription factors in hematopoiesis. Mol Cell Biol 25:1215–1227. 10.1128/MCB.25.4.1215-1227.2005 15684376 10.1128/MCB.25.4.1215-1227.2005 PMC548021

[27] Varghese B, Del Gaudio N, Cobellis G et al (2021) KDM4 involvement in breast cancer and possible therapeutic approaches. Front Oncol 11:750315. 10.3389/fonc.2021.750315 34778065 10.3389/fonc.2021.750315 PMC8581295

[28] Lin CY, Wang BJ, Fu YK et al (2022) Inhibition of KDM4C/c-Myc/LDHA signalling axis suppresses prostate cancer metastasis via interference of glycolytic metabolism. Clin Transl Med 12:e764. 10.1002/ctm2.764 35343073 10.1002/ctm2.764 PMC8958350

[29] Yang JNH, Chen X (2021) GATA1-activated HNF1A-AS1 facilitates the progression of triple-negative breast cancer via sponging miR-32-5p to Upregulate RNF38. Cancer Manag Res 13:1357–1369. 10.2147/CMAR.S274204 33603481 10.2147/CMAR.S274204 PMC7886384

[30] Xie W, Qin W, Qin A et al (2016) GATA1 promotes tumorigenesis and metastasis in breast cancer by cooperating with ZEB2. Int J Clin Exp Pathol 9:4167–4178.

[31] Li CF, Chen JY, Ho YH et al (2019) Snail-induced claudin-11 prompts collective migration for tumour progression. Nat Cell Biol 21:251–262. 10.1038/s41556-018-0268-z 30664792 10.1038/s41556-018-0268-z

[32] Liberzon A, Birger C, Thorvaldsdottir H et al (2015) The molecular signatures database (MSigDB) hallmark gene set collection. Cell Syst 1:417–425. 10.1016/j.cels.2015.12.004 26771021 10.1016/j.cels.2015.12.004 PMC4707969

[33] Nagy A, Munkacsy G, Gyorffy B (2021) Pancancer survival analysis of cancer hallmark genes. Sci Rep 11:6047. 10.1038/s41598-021-84787-5 33723286 10.1038/s41598-021-84787-5 PMC7961001

[34] Liu J, Lichtenberg T, Hoadley KA et al (2018) An integrated TCGA pan-cancer clinical data resource to drive high-quality survival outcome analytics. Cell 173:400-416 e411. 10.1016/j.cell.2018.02.052 29625055 10.1016/j.cell.2018.02.052 PMC6066282

[35] Wu MC, Cheng HH, Li TS et al (2019) KDM4B is a coactivator of c-Jun and involved in gastric carcinogenesis. Cell Death Dis 10:68. 10.1038/s41419-019-1305-y 30683841 10.1038/s41419-019-1305-y PMC6347645

[36] Spassov VZ, Yan L (2008) A fast and accurate computational approach to protein ionization. Protein Sci 17:1955–1970. 10.1110/ps.036335.108 18714088 10.1110/ps.036335.108 PMC2578799

[37] Spassov VZ, Flook PK, Yan L (2008) LOOPER: a molecular mechanics-based algorithm for protein loop prediction. Protein Eng Des Sel 21:91–100. 10.1093/protein/gzm083 18194981 10.1093/protein/gzm083

[38] Chen R, Li L, Weng Z (2003) ZDOCK: an initial-stage protein-docking algorithm. Proteins 52:80–87. 10.1002/prot.10389 12784371 10.1002/prot.10389

[39] Pierce B, Weng Z (2007) ZRANK: reranking protein docking predictions with an optimized energy function. Proteins 67:1078–1086. 10.1002/prot.21373 17373710 10.1002/prot.21373

[40] Shetty T, Sishtla K, Park B et al (2020) Heme synthesis inhibition blocks angiogenesis via mitochondrial dysfunction. IScience 23:101391. 10.1016/j.isci.2020.101391 32755804 10.1016/j.isci.2020.101391 PMC7399258

[41] Tanimura N, Miller E, Igarashi K et al (2016) Mechanism governing heme synthesis reveals a GATA factor/heme circuit that controls differentiation. Embo Rep 17:249–265. 10.15252/embr.201541465 26698166 10.15252/embr.201541465 PMC5290819

[42] Liu JS, Fang WK, Yang SM et al (2022) Natural product myricetin is a pan-KDM4 inhibitor which with poly lactic-co-glycolic acid formulation effectively targets castration-resistant prostate cancer. J Biomed Sci 29:29. 10.1186/s12929-022-00812-3 35534851 10.1186/s12929-022-00812-3 PMC9082844

[43] Marine JC, Dawson SJ, Dawson MA (2020) Non-genetic mechanisms of therapeutic resistance in cancer. Nat Rev Cancer 20:743–756. 10.1038/s41568-020-00302-4 33033407 10.1038/s41568-020-00302-4

[44] Cheng HY, Hsieh CH, Lin PH et al (2022) Snail-regulated exosomal microRNA-21 suppresses NLRP3 inflammasome activity to enhance cisplatin resistance. J Immunother Cancer. 10.1136/jitc-2022-004832 36002186 10.1136/jitc-2022-004832 PMC9413180

[45] Chung CH, Lin CY, Chen CY et al (2023) Ferroptosis signature shapes the immune profiles to enhance the response to immune checkpoint inhibitors in head and neck cancer. Adv Sci 10:e2204514. 10.1002/advs.202204514 10.1002/advs.202204514 PMC10214241 37026630

[46] Ajioka RS, Phillips JD, Kushner JP (2006) Biosynthesis of heme in mammals. Biochim Biophys Acta 1763:723–736. 10.1016/j.bbamcr.2006.05.005 16839620 10.1016/j.bbamcr.2006.05.005

[47] Katsumura KR, Bresnick EH (2017) The GATA factor revolution in hematology. Blood 129:2092–2102. 10.1182/blood-2016-09-687871 28179282 10.1182/blood-2016-09-687871 PMC5391619

[48] Wilkinson-White L, Gamsjaeger R, Dastmalchi S et al (2011) Structural basis of simultaneous recruitment of the transcriptional regulators LMO2 and FOG1/ZFPM1 by the transcription factor GATA1. Proc Natl Acad Sci U S A 108:14443–14448. 10.1073/pnas.1105898108 21844373 10.1073/pnas.1105898108 PMC3167507

[49] Chu CH, Wang LY, Hsu KC et al (2014) KDM4B as a target for prostate cancer: structural analysis and selective inhibition by a novel inhibitor. J Med Chem 57:5975–5985. 10.1021/jm500249n 24971742 10.1021/jm500249n PMC4216216

[50] Wu MJ, Chen CJ, Lin TY et al (2021) Targeting KDM4B that coactivates c-Myc-regulated metabolism to suppress tumor growth in castration-resistant prostate cancer. Theranostics 11:7779–7796. 10.7150/thno.58729 34335964 10.7150/thno.58729 PMC8315051

[51] Soini Y, Kosma VM, Pirinen R (2015) KDM4A, KDM4B and KDM4C in non-small cell lung cancer. Int J Clin Exp Pathol 8:12922–12928 26722485 PMC4680430

[52] Baby S, Gurukkala Valapil D, Shankaraiah N (2021) Unravelling KDM4 histone demethylase inhibitors for cancer therapy. Drug Discov Today 26:1841–1856. 10.1016/j.drudis.2021.05.015 34051367 10.1016/j.drudis.2021.05.015

[53] Wu Q, Young B, Wang Y et al (2022) Recent advances with KDM4 inhibitors and potential applications. J Med Chem 65:9564–9579. 10.1021/acs.jmedchem.2c00680 35838529 10.1021/acs.jmedchem.2c00680 PMC9531573

[54] Rojo de la Vega M, Chapman E, Zhang DD (2018) NRF2 and the Hallmarks of Cancer. Cancer Cell 34:21–43. 10.1016/j.ccell.2018.03.022 29731393 10.1016/j.ccell.2018.03.022 PMC6039250

