# Supplementary material for: KDM4C works in concert with GATA1 to regulate heme metabolism in head and neck squamous cell carcinoma
Source: Cell Mol Life Sci. 2025 Apr 21;82(1):170. doi: 10.1007/s00018-025-05693-x (PMC12011672; doi:10.1007/s00018-025-05693-x)
Supplement: Supplementary file 1 — Supplementary file1 (DOCX 3746 KB) [file 18_2025_5693_MOESM1_ESM.docx]

**Supplementary Materials**

**KDM4C works in concert with GATA1 to regulate heme metabolism in head and neck squamous cell carcinoma**

Meng-Jen Wu^1,†^, Shan-Min Yang^1,†^, Wei-Kai Fang^1^, Tsan-Jan Chen^1^, Chun-Yi Wu^1^, Yen-Jung Hsu^1^, Cheng-En Shen^1^, Yu-Chia Cheng^1^, Wan-Chen Hsieh^1^, Chiou-Hwa Yuh^2^, Muh-Hwa Yang^3^, Hsing-Jien Kung^4,5^, and Wen-Ching Wang^1*^

^1^Institute of Molecular and Cellular Biology and Department of Life Science, National Tsing-Hua University, Hsinchu 30013, Taiwan (R.O.C).

^2^Institute of Molecular and Genomic Medicine, National Health Research Institutes, Miaoli 35053, Taiwan (R.O.C).

^3^Institute of Clinical Medicine, National Yang Ming Chiao Tung University, Taipei 11221, Taiwan

^4^Graduate Institute of Cancer Biology and Drug Discovery, Taipei Medical University, Taipei 11031, Taiwan (R.O.C).

^5^Department of Biochemistry and Molecular Medicine, University of California Davis School of Medicine, University of California Davis Cancer Centre, Sacramento, CA 95817, USA.

^†^These authors contributed equally: Meng-Jen Wu and Shan-Min Yang.

*Correspondence e-mail: wcwang@gapp.nthu.edu.tw; Tel: +886-3-5742766

**Supplementary Figures S1-S8**

**Supplementary Tables S1-S5**

**Supplementary Materials and methods**

**Protein purifications**

**Protein pull down assay**

**
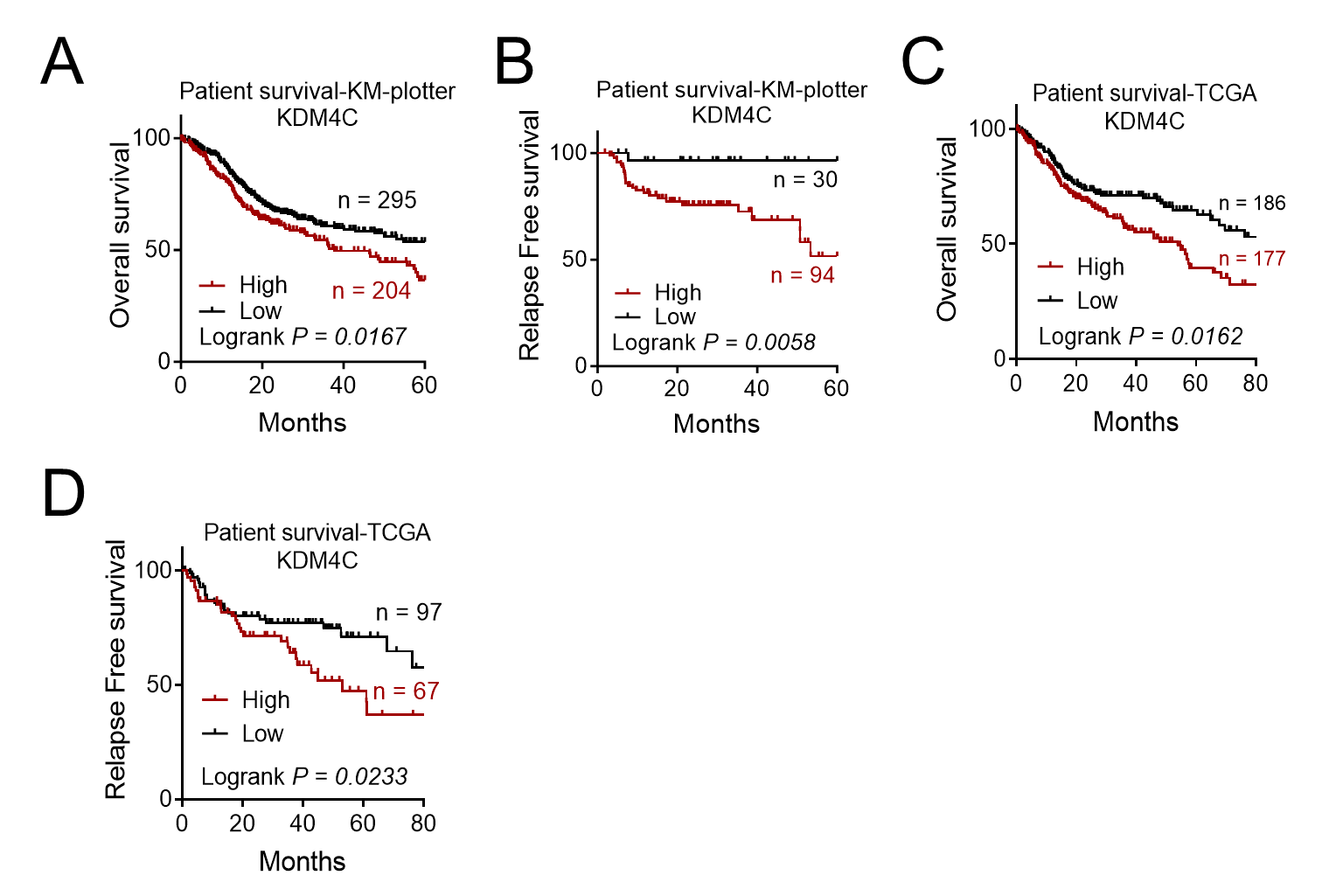
**

**Figure S1.** KDM4C is overexpressed and associated with poor clinical outcomes in HNSCC. **A, B** Survival curves for overall survival (**A**) and relapse-free survival (**B**) of KDM4C in HNSCC patients. The data was analyzed using the KM-plotter database (<https://kmplot.com/analysis/>) with auto-selected best cutoff. **C** The overall survival curve of high and low KDM4C expression groups in TCGA HNSCC patients, grouped using the best cut-off z-score. **D** The relapse-free survival curve of high and low KDM4C expression groups in TCGA HNSCC patients, grouped using the best cut-off z-score.

**
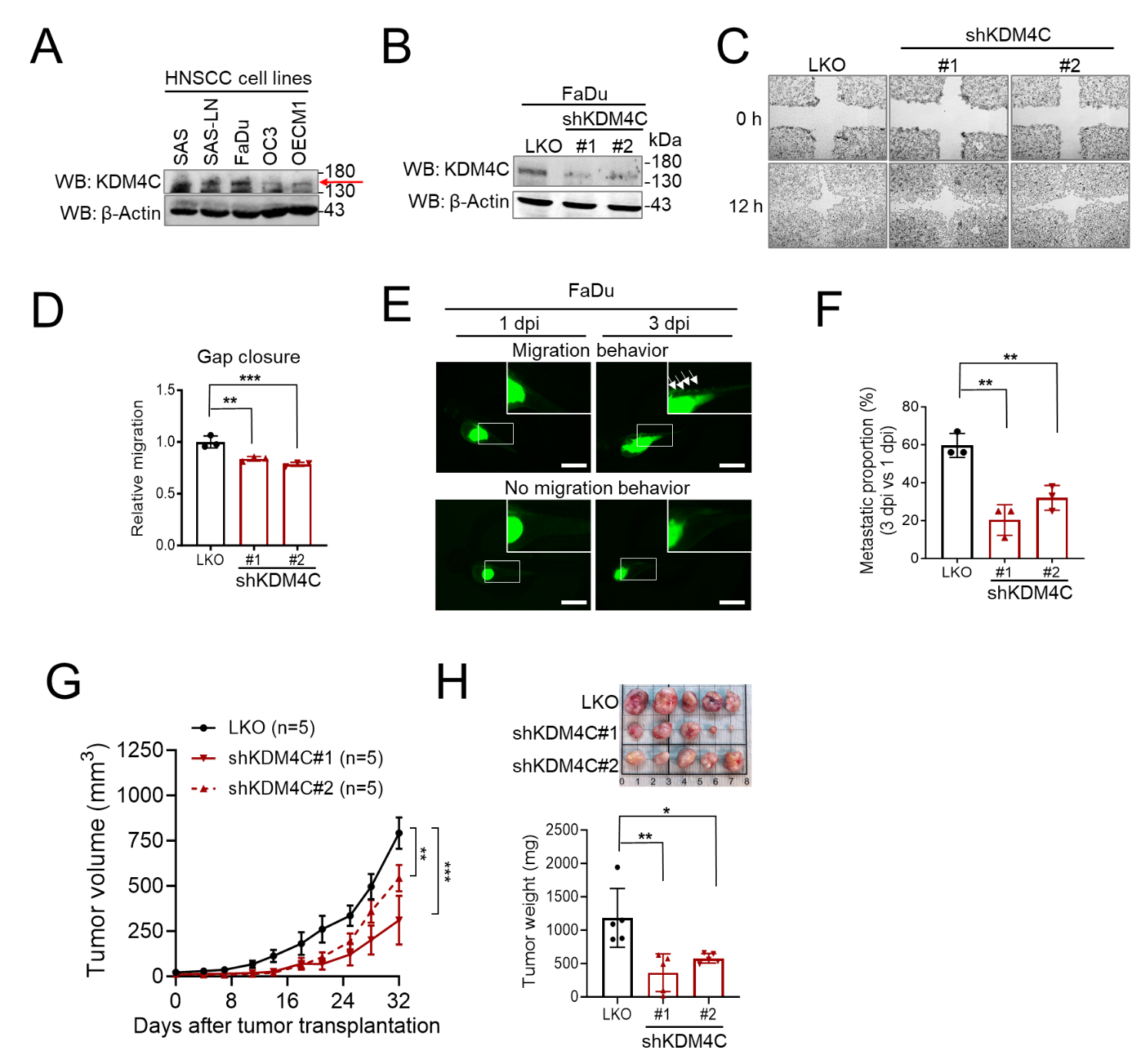
**

**Figure S2.** KDM4C enhances cell migration and is crucial for tumor growth in FaDu Cells. **A** The expression level of KDM4C in various HNSCC cell lines. **B** Generation of KDM4C-knockdown (KDM4C-KD) FaDu cells. Cells were infected with lentivirus carrying control pLKO.1 (LKO), or shKDM4C constructs (#1 or #2), followed by puromycin selection. KDM4C expression was analyzed by Western blot. Actin was used as the internal control. **C** Wound healing assay of LKO and KDM4C-KD FaDu cells. **D** Quantification of the wound closure area from (**C**). **E** Zebrafish xenotransplantation assay of LKO and KDM4C-KD FaDu cells. Representative fluorescence images of zebrafish embryos injected with LKO (control) or KDM4C-knockdown (shKDM4C#1 and shKDM4C#2) FaDu cells at 1 dpi and 3 dpi. White arrows indicate migrating tumor cells. Zebrafish with fluorescence confined to the yolk sac were classified as non-migratory. Scale bar, 1 mm. **F** Quantification of cell migration from (**E**). Each data point represents the percentage of embryos exhibiting tumor cell migration at 3 dpi in one of three independent biological experiments. The total number of embryos analyzed per group is as follows: LKO (n = 27), shKDM4C#1 (n = 25), and shKDM4C#2 (n = 22). **G**, **H** Examination of LKO and KDM4C-KD FaDu cells (1×10^6^ cells) xenografted in BALB/cAnN.Cg-Foxn1nu/CrlNarl mice. Measurements of tumor volume were recorded twice a week (**G**), and recorded tumor weight at the sacrificed endpoint (**H**). Data in (**D**, **F**) are represented in individual points and mean, and data in (**G**) are mean ± SEM. P-values are determined by one-way ANOVA with Tukey’s multiple comparisons test (**D**, **F**, **H**) or two-way ANOVA with Tukey’s multiple comparisons test (**F**). **P* < 0.05, ***P* < 0.01, ****P* < 0.001, ns: not significant

**
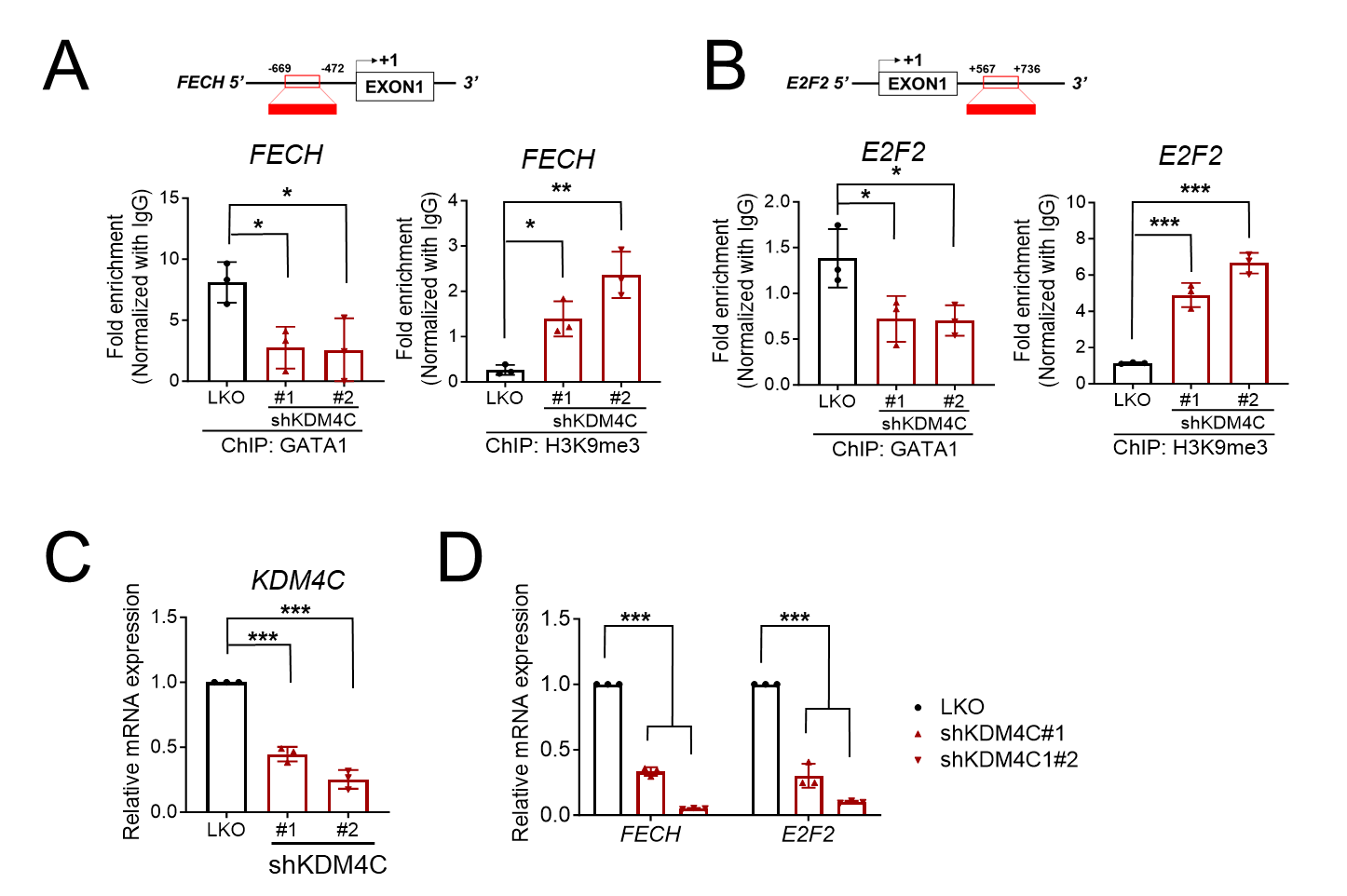
**

**Figure S3.** KDM4C and GATA1 regulate the expression of heme metabolism genes in FaDu cells. **A**, **B** Analysis of occupancy changes at the promoter region of *FECH* (**A**) and *E2F2* (**B**) via ChIP-qPCR. ChIP was conducted with specific antibodies (anti-GATA1 and anti-H3K9me3) in LKO and KDM4C-KD FaDu cells. **C**, **D** Relative mRNA levels of heme-metabolism genes (*FECH* and *E2F2*) in the LKO and KDM4C-KD FaDu cells. Data in (**A**–**D**) are represented in individual points and mean. *P*-values are determined by one-way ANOVA with Tukey’s multiple comparisons test (**A**–**C**), or two-way ANOVA with Tukey’s multiple comparisons test (**D**). **P* < 0.05, ***P* < 0.01, ****P* < 0.001, ns: not significant

**
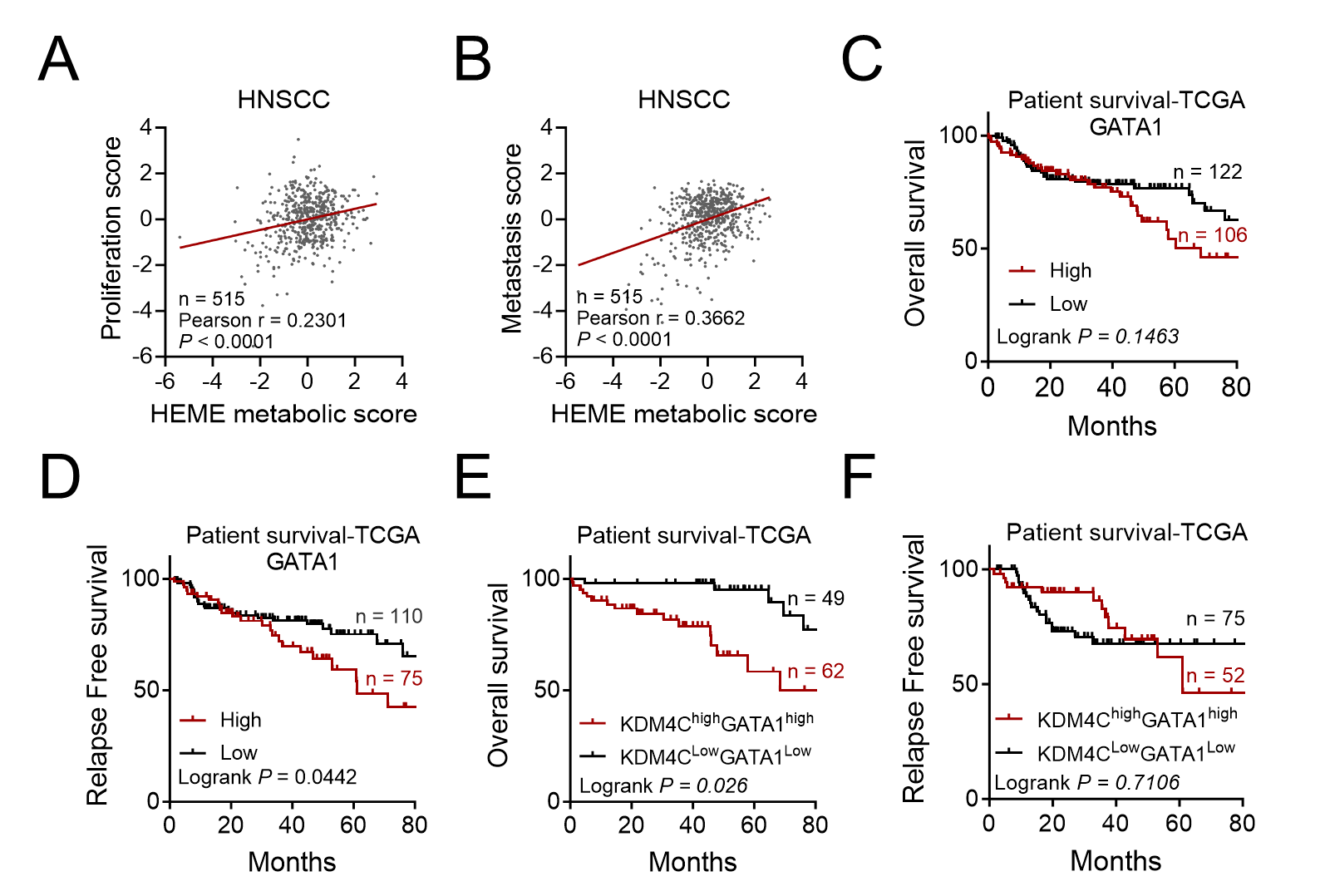
**

**Figure S4. Correlation of heme metabolism with proliferation, metastasis, and clinical outcomes in HNSCC. A**, **B** Heme metabolism, proliferation, and metastasis scores were calculated using single-sample Gene Set Enrichment Analysis (ssGSEA) (z-score) from the HNSCC TCGA dataset. Pearson correlation analysis reveals a positive correlation between heme metabolism scores and metastasis scores (**A**) and between heme metabolism scores and proliferation scores (**B**). The gene sets used for these scores are sourced from GSEA, specifically Hallmark \HEME metabolism, GOBP REGULATION OF EPITHELIAL CELL PROLIFERATION, and BIDUS METASTASIS UP. **C**, **D** Kaplan-Meier survival analysis of HNSCC patients from TCGA, comparing overall survival (**C**) and relapse-free survival (**D**) between high and low GATA1 expression groups. **E, F** Survival analysis of HNSCC patients stratified by KDM4C and GATA1 co-expression levels, showing overall survival (**E**) and relapse-free survival (**F**) for KDM4C^high^/GATA1^high^ versusKDM4C^low^/GATA1^low^ groups. Patient grouping was determined using the best cut-off Z-core.

**
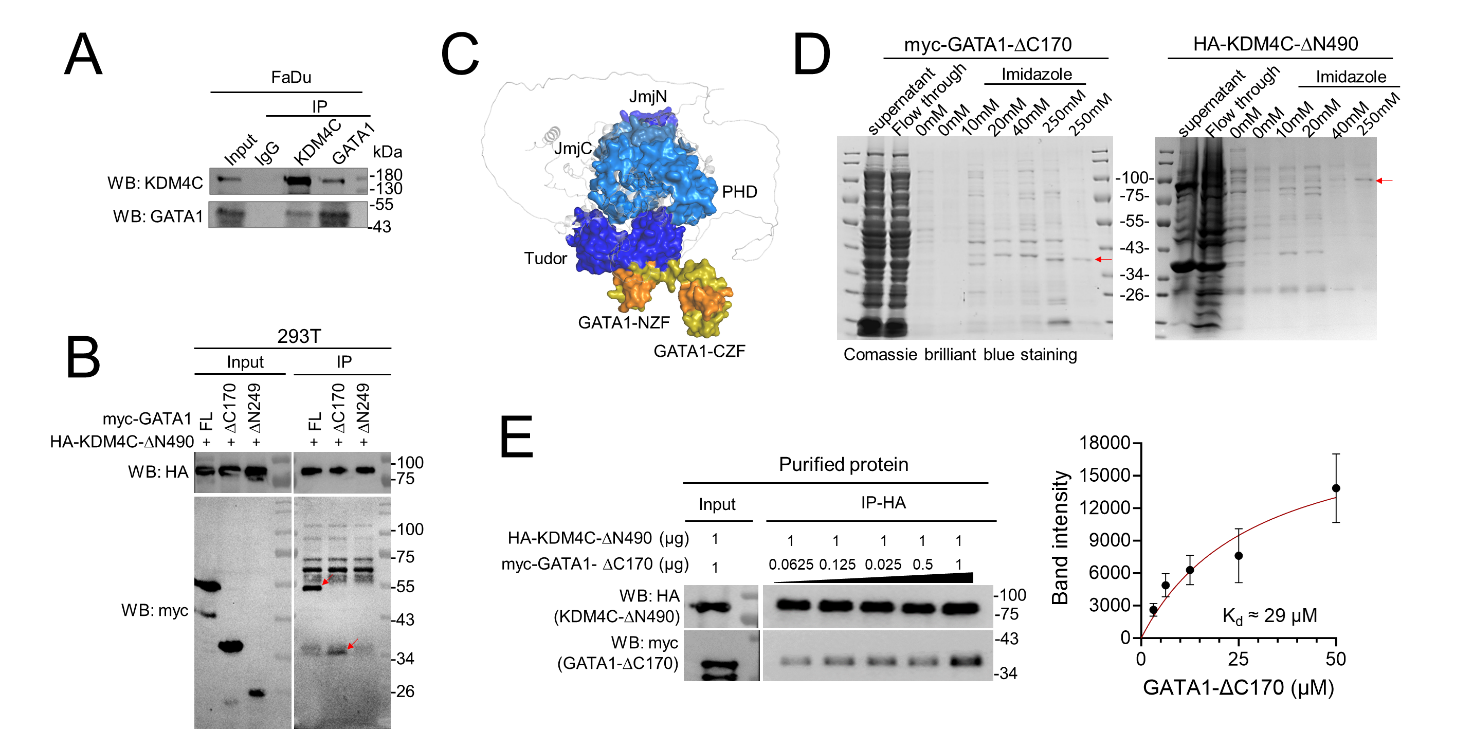
**

**Figure S5.** Interaction Analysis of KDM4C and GATA1 in HNSCC Cells. **A** Western blot analysis of endogenous immunoprecipitation (IP) in FaDu cells. IP was performed using anti-KDM4C or anti-GATA1 antibodies, with rabbit IgG as a negative control. The blot demonstrates the specific interaction between KDM4C and GATA1. **B** Co-immunoprecipitation (Co-IP) assay conducted with an anti-HA antibody in lysates from HEK293T cells co-transfected with HA-tagged KDM4C-∆N490 vector and one of the myc-tagged constructs: mock, full-length (FL) GATA1, or GATA1 truncated mutants (∆C170 and ∆N249). Western blot analysis shows the specific interaction between KDM4C and the GATA1 constructs. **C** Docking analysis of the KDM4C and GATA1 interaction using BIOVIA Discovery Studio 2022. The model of KDM4C (AlphaFold ID: AF-Q9H3R0-F1) includes JmjN, JmjC, PHD, and Tudor domains (in blue), while the GATA1 segment (AlphaFold ID: AF-P15976-F1) consists of N-terminal (NZF) and C-terminal (CZF) zinc finger domains (in orange), indicating key interaction sites. **D** Protein purification process for KDM4C-∆N490 and GATA1-∆C170, visualized using Coomassie Brilliant Blue staining. The bands confirm the successful purification of the proteins used in subsequent assays. **E** In vitro pull-down assay using purified KDM4C-∆N490 and GATA1-∆C170. Increasing amounts of GATA1-∆C170 (0.0625, 0.125, 0.25, 0.5, and 1 μg) were incubated with a constant amount of KDM4C-∆N490 (1 μg). The interaction was detected by western blot, demonstrating concentration-dependent binding. The band intensity of Myc-GATA1-∆C170 was measured by ImageJ analysis. The binding affinity between KDM4C-∆N490 and GATA1-∆C170 was quantitively estimated using the one-site specific binding equation (Y = Bmax*X/(Kd + X).

**
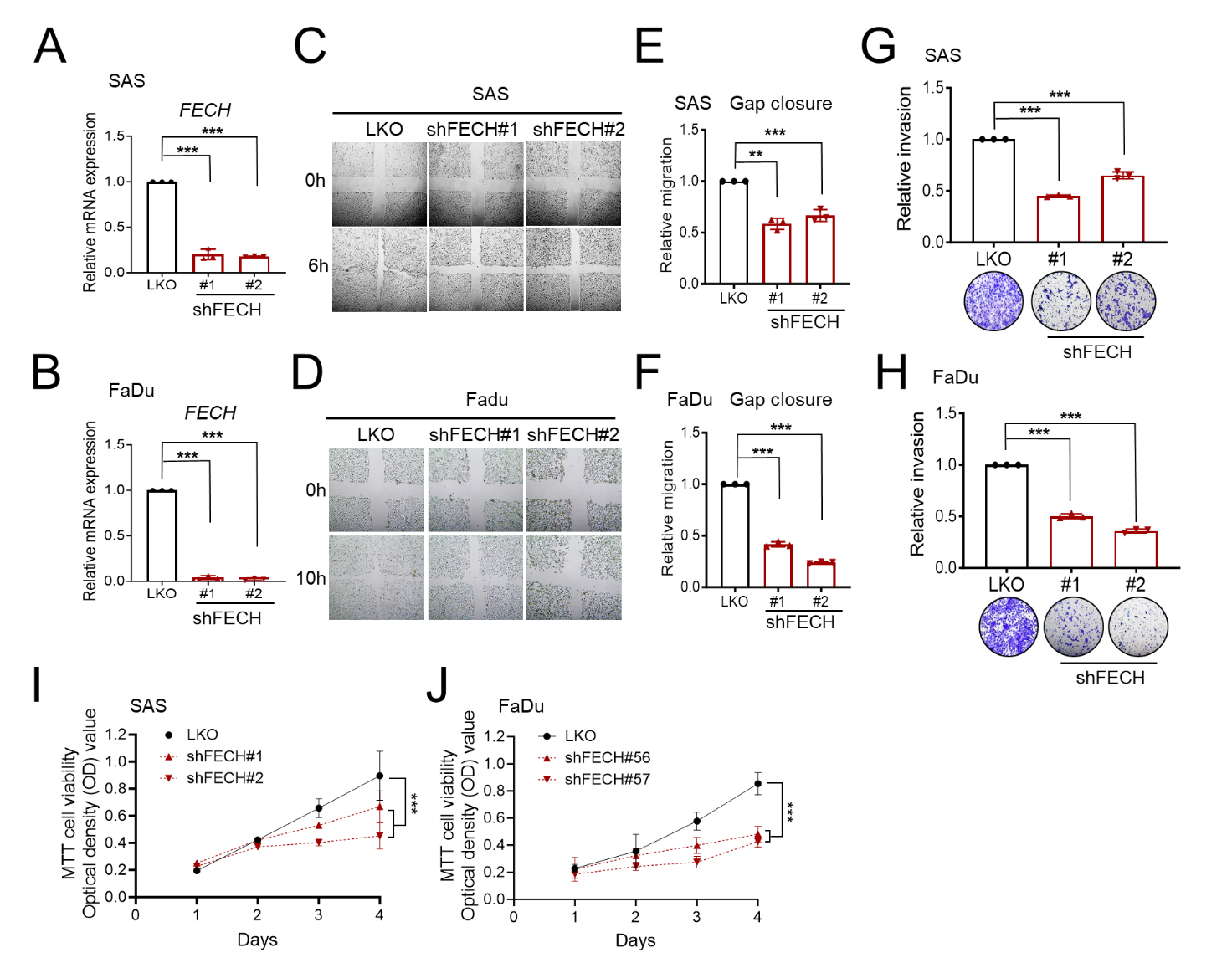
**

**Figure S6.** Effect of FECH knockdown on cell migration, invasion, and proliferation in SAS and FaDu cells. **A**, **B** Generation of FECH-knockdown (KD) SAS (**A**) and FaDu (**B**) cells. Cells were infected with lentivirus carrying control pLKO.1 (LKO), or shFECH constructs (#1 or #2), followed by puromycin selection. *FECH* expression was analyzed using qRT-PCR. **C**, **D** Wound healing assay of LKO and FECH-KD SAS (**C**) and FaDu (**D**) cells. **E**, **F** Quantification of the wound closure area from (**C, D**). **G**, **H** Invasion assay of LKO and FECH-KD SAS (**G**) and FaDu (**H**) cells. **I**, **J** MTT cell proliferation assay of LKO and FECH-KD SAS (**I**) and FaDu (**J**) cells at indicated time points. Data in (**A**, **B**, **E**–**H**) are represented in individual points and mean, and data in (**I**, **J**) are mean ± SD. P-values are determined by one-way ANOVA with Tukey’s multiple comparisons test (**A**, **B**, **E**–**H**) and two-way ANOVA with Tukey’s multiple comparisons test (**I**, **J**). **P* < 0.05, ***P* < 0.01, ****P* < 0.001, ns: not significant

**
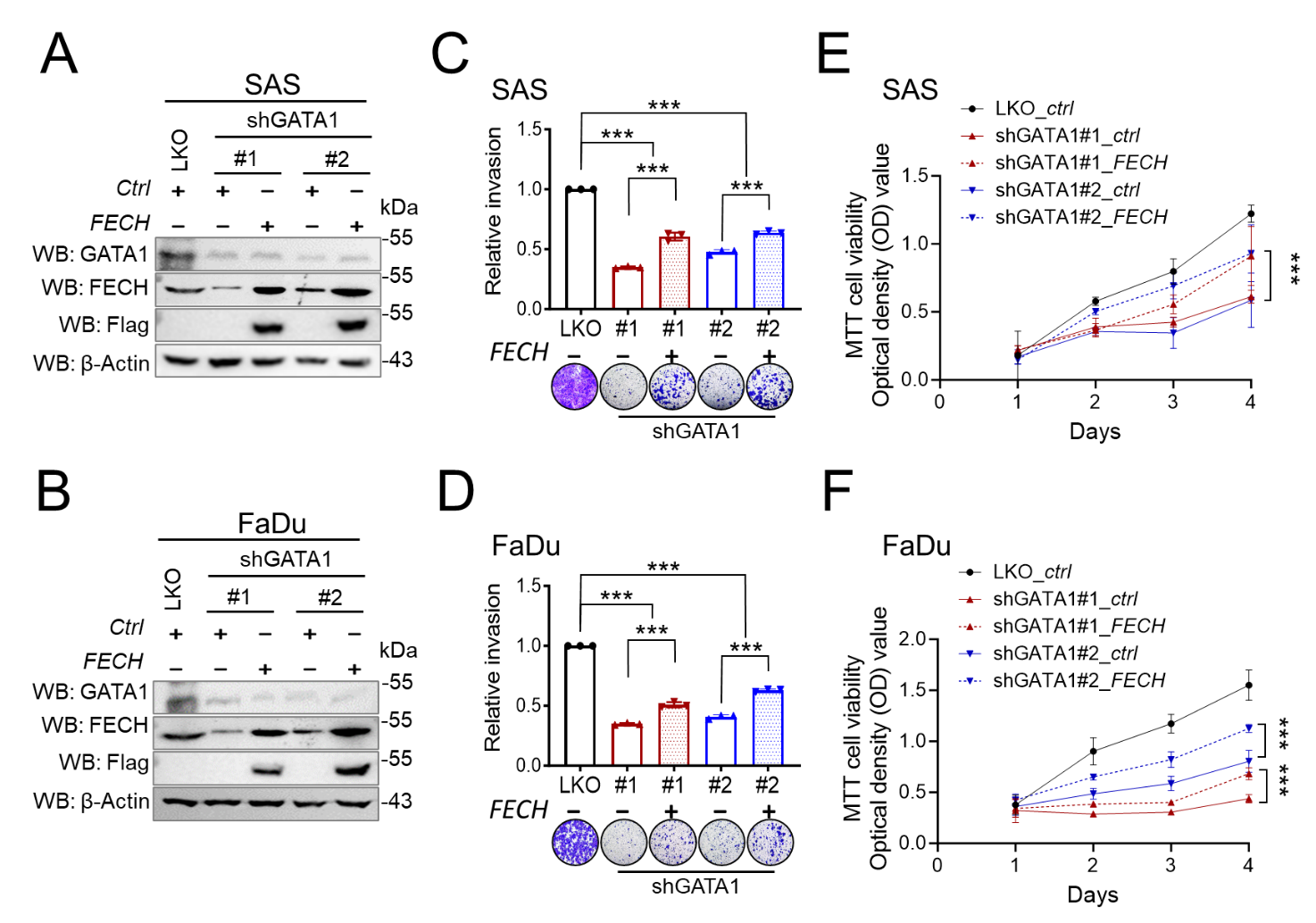
**

**Figure S7.** Effect of FECH overexpression on invasion and proliferation in GATA1-knockdown SAS and FaDu cells. **A, B** Analysis of FECH expression in LKO*,* or shGATA1 constructs (shGATA1#1 or #2) SAS (**A**) and FaDu (**B**) cells. Cells were transfected with control or Flag-FECH expression vector, followed by Western blot analysis. **C**, **D** Invasion assay of LKO and FECH-overexpressing GATA1-KD SAS (**C**) and FaDu (**D**) cells. **E**, **F** MTT cell proliferation assay of LKO and FECH-overexpressing GATA1-KD SAS (**E**) and FaDu (**F**) cells at indicated time points. Data in (**C**, **D**) are represented in individual points and mean, and data in (**E**, **F**) are mean ± SD. P-values are determined by one-way ANOVA with Tukey’s multiple comparisons test (**C**, **D**) and two-way ANOVA with Tukey’s multiple comparisons test (**E**, **F**). **P* < 0.05, ***P* < 0.01, ****P* < 0.001, ns: not significant

**
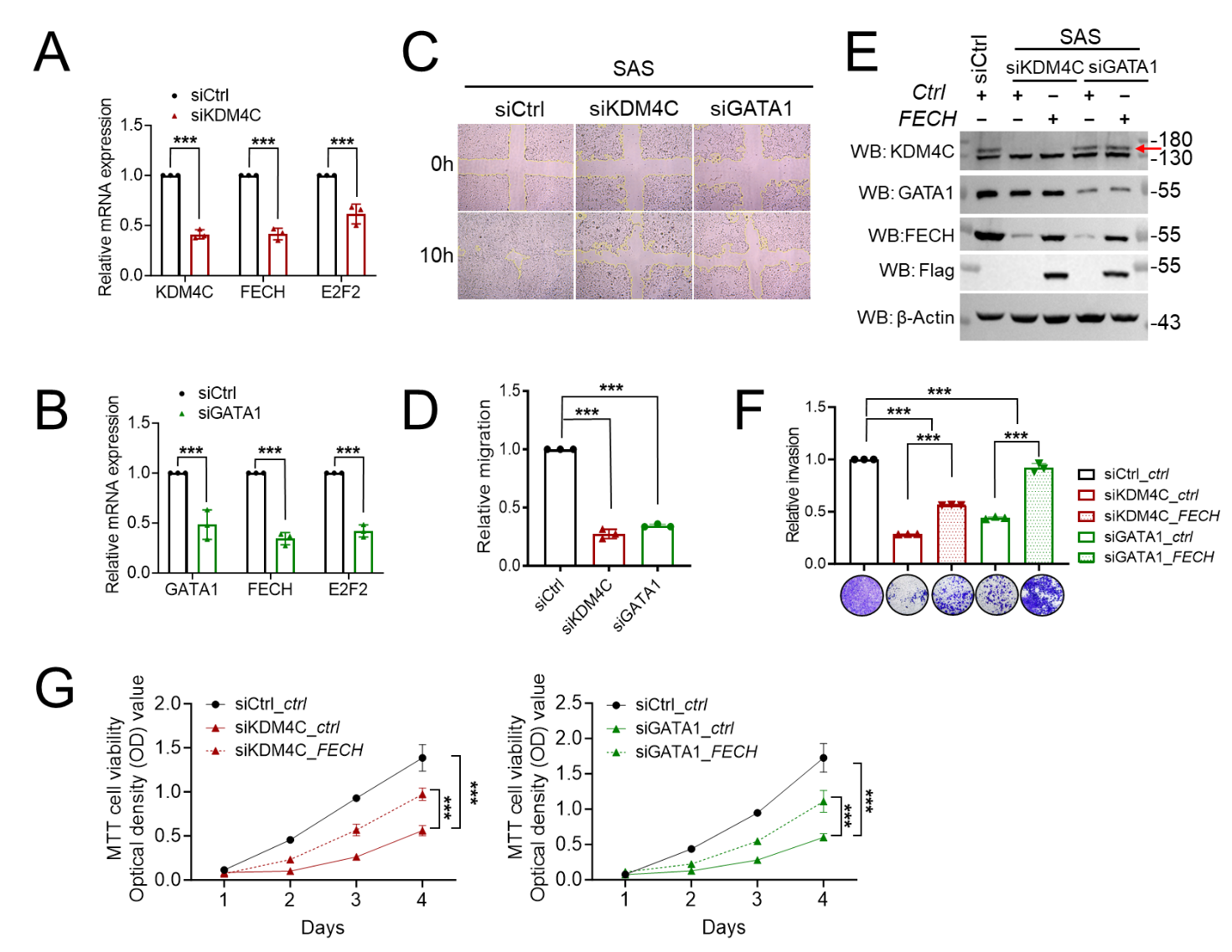
**

**Figure S8.** Effects of siRNA-mediated knockdown of KDM4C or GATA1 on downstream gene expression (*FECH* and *E2F2*), cell proliferation, and migration in SAS cells, with phenotype restoration upon FECH overexpression. **A**, **B** qRT-PCR analysis of FECH and E2F2 expression levels in SAS cells transfected with control siRNA (siCtrl), KDM4C siRNA (siKDM4C) (**A**), or GATA1 siRNA (siGATA1) (**B**). **C** Representative images from the wound healing assay of siCtrl, KDM4C-KD (siKDM4C), and GATA1-KD (siGATA1) SAS cells. **D** Quantification of wound closure from (**C**). **E** Western blot analysis of *FECH* expression in siCtrl, KDM4C-KD (siKDM4C), and GATA1-KD (siGATA1) SAS cells transfected with either a control or Flag-FECH expression vector. **F** Invasion assay of siCtrl and FECH-overexpressing cells in KDM4C-KD (siKDM4C) or GATA1-KD (siGATA1) SAS cells. **G** MTT cell proliferation assay of siCtrl and FECH-overexpressing cells in KDM4C-KD (left, siKDM4C) and GATA1-KD (right, siGATA1) SAS cells over time. Data in (**A**, **B**, **D**, **F**) are shown as individual points with mean, and data in (**G**) as mean ± SD. P-values were determined using one-way ANOVA with Tukey’s multiple comparisons test (**F**) and two-way ANOVA with Tukey’s multiple comparisons test (**A, B, F, G**). **P* < 0.05, ***P* < 0.01, ****P* < 0.001, ns: not significant

**
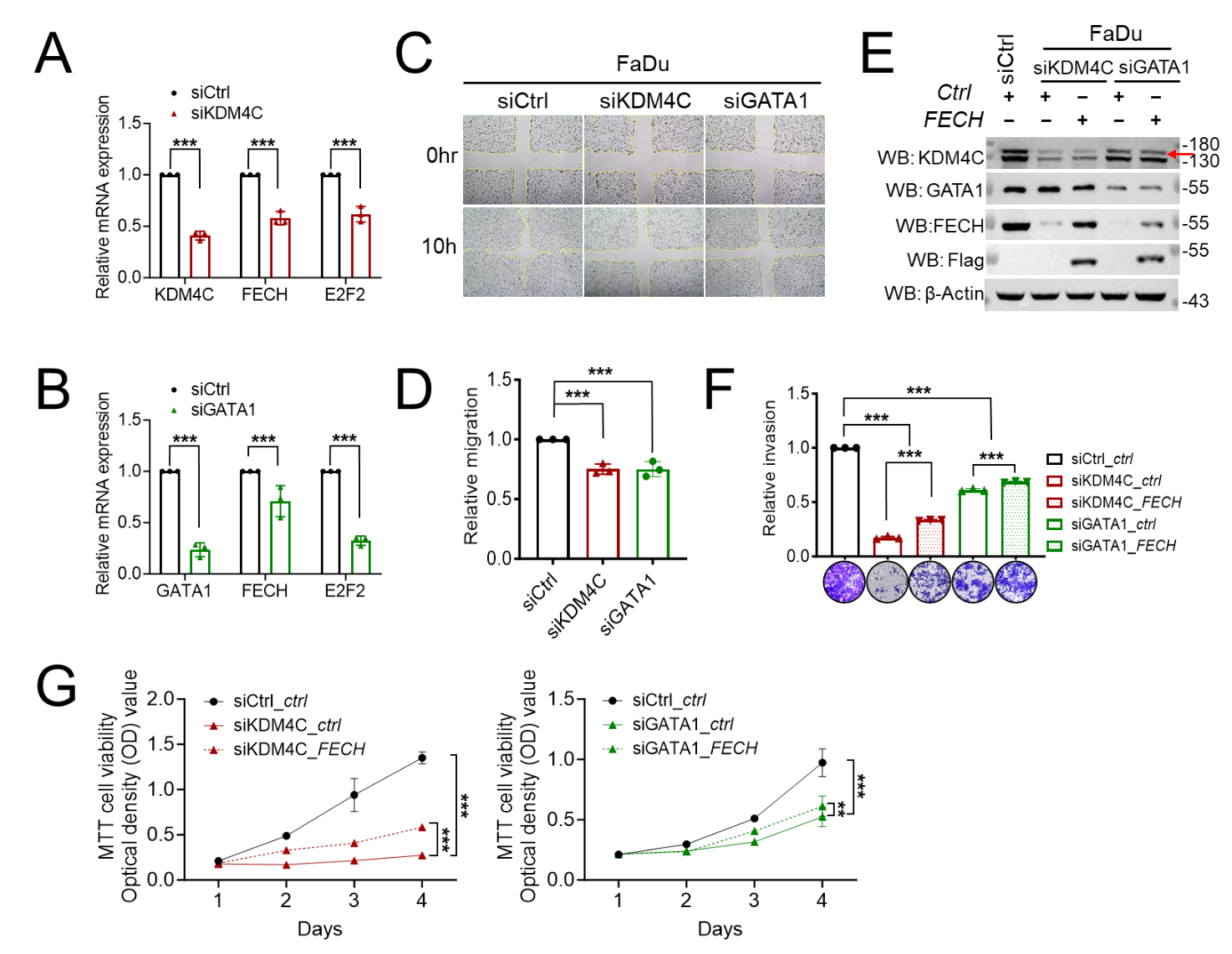
**

**Figure S9.** Effects of siRNA-mediated knockdown of KDM4C or GATA1 on downstream gene expression (*FECH* and *E2F2*), cell proliferation, and migration in FaDu cells, with phenotype restoration upon FECH overexpression. **A**, **B** qRT-PCR analysis of *FECH* and *E2F2* expression in FaDu cells transfected with control siRNA (siCtrl), KDM4C siRNA (siKDM4C) (**A**), or GATA1 siRNA (siGATA1) (**B**). **C** Representative images from the wound healing assay of siCtrl, KDM4C-KD (siKDM4C), and GATA1-KD (siGATA1) FaDu cells. **D** Quantification of wound closure from (C). **E** Western blot analysis of *FECH* expression in siCtrl, KDM4C-KD (siKDM4C), and GATA1-KD (siGATA1) FaDu cells transfected with either a control or Flag-FECH expression vector. **F** Invasion assay of siCtrl and FECH-overexpressing cells in KDM4C-KD (siKDM4C) or GATA1-KD (siGATA1) FaDu cells. **G** MTT cell proliferation assay of siCtrl and FECH-overexpressing cells in KDM4C-KD (left, siKDM4C) and GATA1-KD (right, siGATA1) FaDu cells over time. Data in (**A**, **B**, **D**, **F**) are shown as individual points with mean, and data in (**G**) as mean ± SD. *P*-values were determined using one-way ANOVA with Tukey’s multiple comparisons test (**F**) and two-way ANOVA with Tukey’s multiple comparisons test (**A, B, F, G**). **P* < 0.05, ***P* < 0.01, ****P* < 0.001, ns: not significant

**
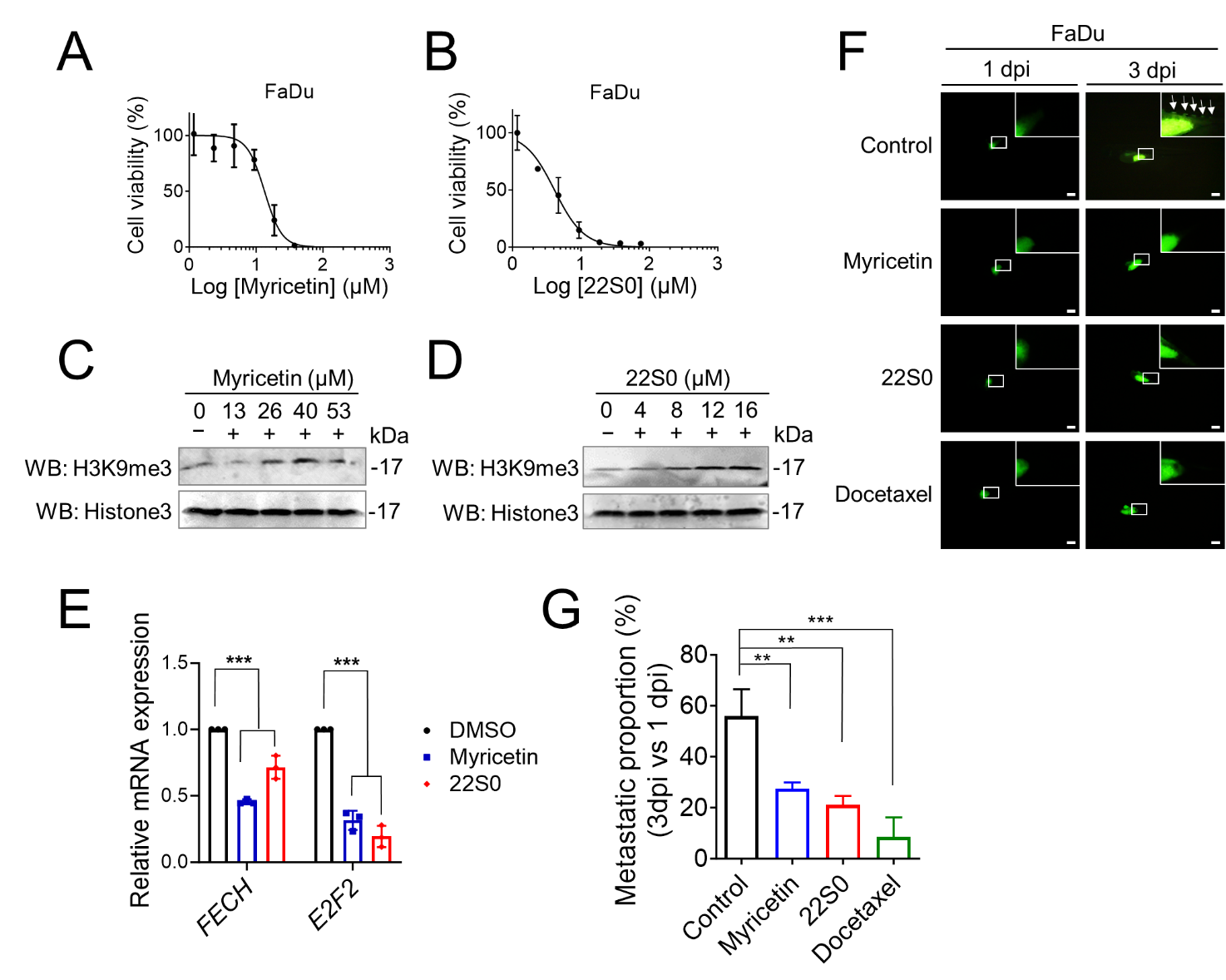
**

**Figure S10.** Effects of KDM4 inhibitors on cell survival, H3K9me3 levels, heme metabolism gene expression, and cell migration in HNSCC cells. **A**, **B** FaDu were treated with different concentrations of myricetin (**A**) or 22S0 (**B**) for 3 days. The relative cell survival rates were measured using the MTT assay. Cell viability was determined and shown as dose-response curves. **C**, **D** Analysis of H3K9me3 levels in FaDu cells treated with a selected concentration of inhibitors for 3 days. **E** The relative mRNA levels of heme-metabolism genes (*FECH* and *E2F2*) in FaDu cells following 24-hour treatment with control (DMSO, 0.1%), myricetin (12.5 µM), or 22S0 (3.125 µM). **F** Representative images of FaDu cells xenografted in zebrafish subjected to drug treatments. From top to bottom, treatments include DMSO 0.1% (as control), myricetin (13.37 µM), 22S0 (4.11 µM), and docetaxel (0.42 nM). Scale bar: 200 µm. **G** Quantification of cell migration of FaDu cells under various drug treatments from (**F**). Each data point represents the percentage of embryos exhibiting tumor cell migration at 3 dpi in one of three independent biological experiments. The total number of embryos analyzed per group is as follows: control (n = 34), myricetin (n = 34), 22S0 (n = 33), and docetaxel (n = 32). Data in (**E**, **G**) are represented as individual points and mean, and data in (**A**, **B**) are represented in mean ± SD. P-values are determined by two-way ANOVA with Tukey’s multiple comparisons test (**E**) and one-way ANOVA with Tukey’s multiple comparisons test (**G**). **P* < 0.05, ***P* < 0.01, ****P* < 0.001, ns: not significant

**
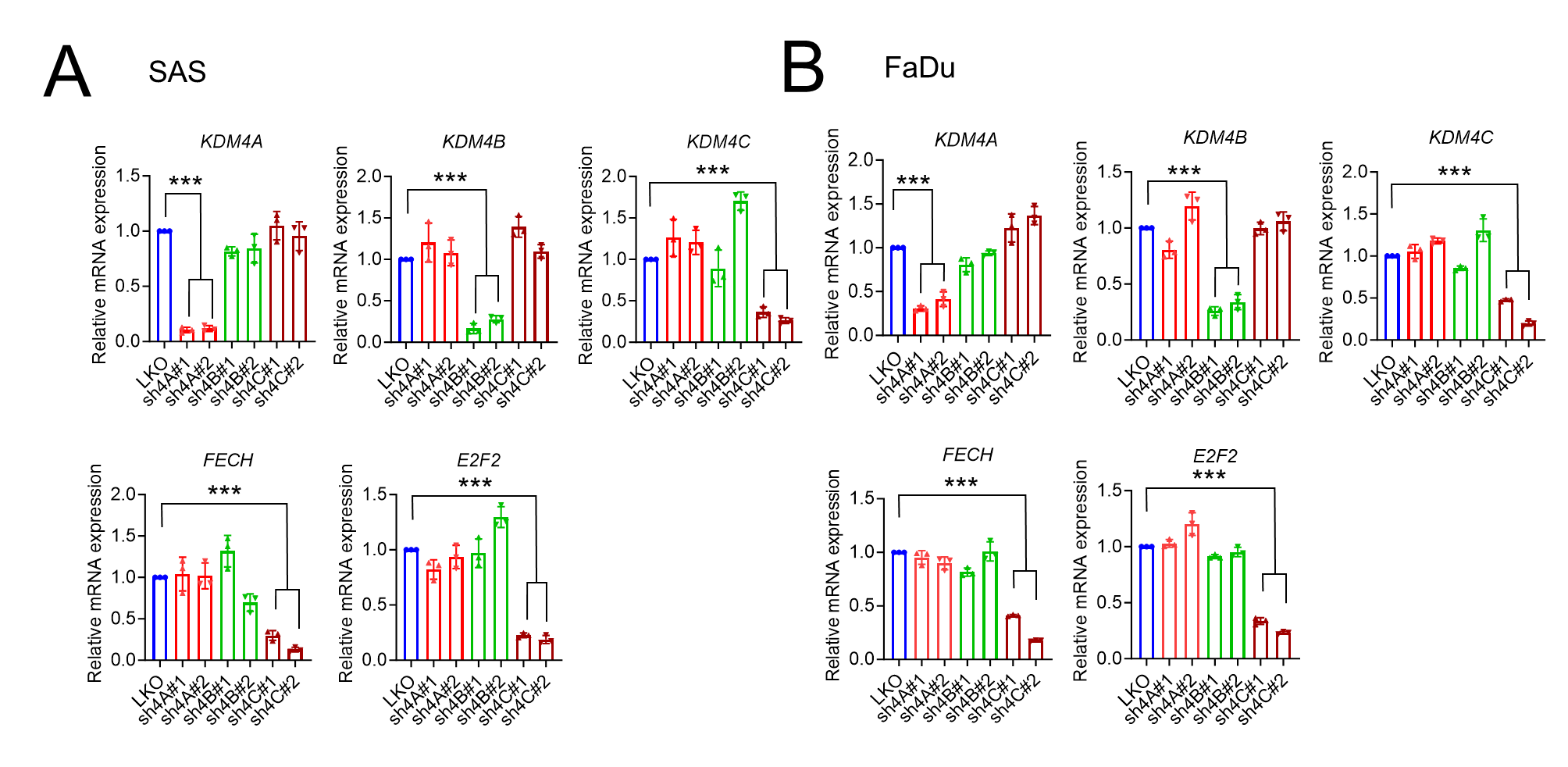
**

**Figure S11.** Depletion of KDM4C, but not KDM4A and KDM4B, reduces the expression of *FECH* and *E2F2*. **A**, **B** The relative mRNA levels of KDM4-knockdown SAS **(A)** and FaDu **(B)** cells. Cells were infected with lentivirus carrying control pLKO.1 (LKO), shKDM4A (sh4A#1 or sh4A#2), shKDM4B (sh4B#1 or sh4B#2), or shKDM4C (sh4C#1 or sh4C#2). Total RNA is prepared and subjected to qRT-PCR for *KDM4A*, *KDM4B*, *KDM4C*, *FECH* and *E2F2* expression. All data are represented as individual points and mean. P-values were calculated by one-way ANOVA with Dunnett’s multiple comparisons test. **P* < 0.05, ***P* < 0.01, ****P* < 0.001, ns: not significant

**Supplementary Table S1: List of antibodies**

| Protein | Vendor | Catalogue Number | Species | Titer | Assay |
| --- | --- | --- | --- | --- | --- |
| KDM4C | Novus Biologicals | NBP1-49600 | Rabbit | 1:1000 | WB |
|  |  |  |  | 1:500 | IP |
|  |  |  |  | 1:100 | ChIP |
|  |  |  |  | 1:100 | IHC |
| GATA1 | Abcam | 181544 | Rabbit | 1:1000 | WB |
|  |  |  |  | 1:500 | IP |
|  |  |  |  | 1:100 | ChIP |
|  |  |  |  | 1:100 | IHC |
|  | Cell Signaling | 3535 | Rabbit | 1:100 | WB |
| H3K9me3 | Active Motif | 39766 | Rabbit | 1:1000 | WB |
|  |  |  |  | 1:100 | ChIP |
| FECH | GeneTex | GTX113435 | Rabbit | 1:1000 | WB |
| beta-Actin | Novus Biologicals | NB600-501 | Mouse | 1:50000 | WB |
| HA-tag | Cell Signaling | 3724 | Rabbit | 1:2000 | WB |
|  |  |  |  | 1:500 | IP |
| Myc-tag | Cell Signaling | 2276 | Mouse | 1:2000 | WB |
|  |  |  |  | 1:500 | IP |
| Flag-tag | Elabscience | E-AB-48025 | Mouse | 1:2000 | WB |
| Rabbit IgG | Merk | PP64 | Rabbit | 1:500 | IP |
| Mouse IgG | Merk | 12-371 | Mouse | 1:500 | IP |
| anti-Rabbit IgG HRP | Abcam | ab6721 | Goat | 1:5000 | WB |
| anti-Mouse IgG HRP | Bethyl Laboratories | A90-116P | Goat | 1:5000 | Wb |
| Veriblot (HRP for IP) | Abcam | ab131366 |  | 1:5000 | WB |

**Supplementary Table S2: Sequences and sources of shRNAs and siRNAs**

| shRNA | Target sequence | Clone ID | SOURCE |
| --- | --- | --- | --- |
| KDM4C#1 | ATACTTGGATTACGAAGATTT | TRCN0000235047 | National RNAi Core Facility, Academia Sinica, Taiwan |
| KDM4C#2 | GCTATGAGAAGCCCGAGAAAT | TRCN0000235048 | National RNAi Core Facility, Academia Sinica, Taiwan |
| GATA1#1 | TGTACCCATTGCTCAACTGTA | TRCN0000019221 | National RNAi Core Facility, Academia Sinica, Taiwan |
| GATA1#2 | ACCATGCGGAAGGATGGTATT | TRCN0000019222 | National RNAi Core Facility, Academia Sinica, Taiwan |
| FECH#1 | GACCATATTGAAACGCTGTAT | TRCN0000083656 | National RNAi Core Facility, Academia Sinica, Taiwan |
| FECH#2 | CAGGGAGACTAAATCCTTCTT | TRCN0000083657 | National RNAi Core Facility, Academia Sinica, Taiwan |
| KDM4A#1 | GACTGCTGTTTATGCTCATTA | TRCN0000234912 | National RNAi Core Facility, Academia Sinica, Taiwan |
| KDM4A#2 | TAGTGAAAGGACGAGCCATTT | TRCN0000234914 | National RNAi Core Facility, Academia Sinica, Taiwan |
| KDM4B#1 | GTGGAAGCTGAAATGCGTGTA | TRCN0000018016 | National RNAi Core Facility, Academia Sinica, Taiwan |
| KDM4B#2 | ACTGAGCAACCTTTGAGATTG | TRCN0000379460 | National RNAi Core Facility, Academia Sinica, Taiwan |
| siRNA | Target sequence | siRNA code | SOURCE |
| siKDM4C | AUUACAUGCUUUCGACAUA | J-004293-09 | Dharmacon |
|  | ACGAAGAUUUGGAGCGCAA | J-004293-10 | Dharmacon |
|  | GCAUAUAUGAUGAGGGUGU | J-004293-11 | Dharmacon |
|  | GAGAAGUCGUCCAAGUCAA | J-004293-12 | Dharmacon |
| siGATA1 | GGACAGGCCACUACCUAUG | J-009610-05 | Dharmacon |
|  | ACGCUGAGGCCUACAGACA | J-009610-06 | Dharmacon |
|  | GCUGGUGGCUUUAUGGUGG | J-009610-07 | Dharmacon |
|  | CCAAGAAGCGCCUGAUUGU | J-009610-08 | Dharmacon |

**Supplementary Table S3: Primer sequences used in this study**

| Primer name | Sequence (5’ to 3’) |
| --- | --- |
| Cloning | |
| GATA1-FL | F: AAAGCGGCCGCATGGAGTTCCCTGGCCTG |
|  | R: CAAGGTACCTGAGCTGAGCGGAGCCACC |
| GATA1-∆N249-F | F: AAAGCGGCCGCATGGTCAGTAAACGGGCAG |
| GATA1-∆C170-R | R: CGGGGTACCCAGGCGCTTCTTGGG |
| GATA1-∆N199-F | R: AAAGCGGCCGCATGGAGGCCAGGGAG |
| KDM4C-FL | F: CAAAGGTACCATGGAGGTGGCCGAG |
|  | R: CTTTCTCGAGCTACTGTCTCTTCTGG |
| KDM4C-ΔN135-F | F: CCAGGTACCGCAGATATTAATGGG |
| KDM4C-ΔN490-F | F: CACTGGTACCTCTAGTGGCTATGAG |
| KDM4C-ΔC185-R | R: CTTTCTCGAGCTACTTCACGTTGGG |
| FECH-F | F: GAATTCGGTACCATGCGTTCACTCGGCGCA |
| FECH-R | R: ATGCAACTCGAGTCACAGCTGCTGGCTGGTG |
| pET28-GATA1-myc-R | R: AAACTCGAGGTCGACGGCGCTATTCAGATC |
| qPCR | |
| KDM4C | F: ACGCGAGTATCTTTCCCCTC |
|  | R: CGCGCTGTGGTTAACTTAGG |
| KDM4A | F: GCCGCTAGAAGTTTCAGTGAG |
|  | R: GCGTCCCTTGGACTTCTTATT |
| KDM4B | F: CTTCACGCAGTACAATATCC |
|  | R: CGTCGTCATCATACAAAGAG |
| GATA1 | F: CTCAATTCAGCCTATTC |
|  | R: GCCCATTCATCTTGTGATAG |
| 18S | F: CGGCTACCACATCCAAGGAA |
|  | R: GCTGGAATTACCGCGGCT |
| ANK1 | F: CCAAGAAGATCATTCGCAAG |
|  | R: GTAATCAATATCGACCTCCAG |
| E2F2 | F: CAACTTTAAGGAGCAGACAG |
|  | R: TATATCTGCAGGTTGTCCTC |
| ENDOD1 | F: AGCAAGACACAGAGAAAATG |
|  | R: AAAGCTACTACTTCCCTCAG |
| FECH | F: GGAAGAATATCCTCTTGGTTC |
|  | R: CACTCCTTGGCTAAAACTTG |
| ChIP-qPCR | |
| E2F2 | F: CCTACTACACACCGCTGTACC |
|  | R: ACCTGGAAAAGCATAGGGGG |
| FECH | F: GACGGAGGTGTAGGAATGAAAT |
|  | R: ACAGGTGAAACTCACTGAAGAG |

**Supplementary Table S4:** **The overall survival rates of all histone demethylases**

| Gene* | No. of cases | HR | 95% CI | *p*-value |
| --- | --- | --- | --- | --- |
| KDM1A | 499 | 1.49 | 1.09-2.05 | 0.013 |
| KDM1B | 499 | 0.80 | 0.60-1.08 | 0.147 |
| KDM2A | 499 | 0.77 | 0.58-1.02 | 0.067 |
| KDM2B | 499 | 0.47 | 0.33-0.66 | 0.001 |
| KDM3A | 499 | 0.73 | 0.52-1.02 | 0.061 |
| KDM3B | 499 | 1.19 | 0.85-1.66 | 0.300 |
| JMJD1C | 499 | 0.76 | 0.56-1.02 | 0.064 |
| KDM4A | 499 | 0.69 | 0.52-0.91 | 0.009 |
| KDM4B | 499 | 0.66 | 0.49-0.89 | 0.006 |
| KDM4C | 499 | 1.4 | 1.06-1.86 | 0.017 |
| KDM4D | 499 | 0.72 | 0.52-1.01 | 0.058 |
| KDM4E | 499 | 0.62 | 0.46-0.83 | 0.001 |
| KDM5A | 499 | 1.19 | 0.86-1.63 | 0.295 |
| KDM5B | 499 | 1.21 | 0.91-1.60 | 0.180 |
| KDM5C | 499 | 1.29 | 0.97-1.72 | 0.079 |
| KDM5D | 499 | 0.58 | 0.43-0.76 | 0.001 |
| KDM6A | 499 | 0.69 | 0.49-0.97 | 0.029 |
| KDM6B | 499 | 1.27 | 0.96-1.68 | 0.088 |
| UTY | 499 | 0.52 | 0.38-0.71 | 0.001 |
| KDM7A | 499 | 0.72 | 0.53-0.97 | 0.031 |
| PHF8 | 499 | 0.79 | 0.59-1.06 | 0.121 |
| PHF2 | 499 | 0.79 | 0.60-1.05 | 0.102 |
| KDM8 | 499 | 0.76 | 0.57-1.00 | 0.053 |
| RIOX1 | 499 | 1.21 | 0.91-1.61 | 0.194 |
| RIOX2 | 499 | 1.61 | 1.15-2.25 | 0.005 |

*Patients are divided based on best cutoff and follow-up thresholds with a 60-month follow-up period.

| Gene* | No. of cases | HR | 95% CI | *p*-value |
| --- | --- | --- | --- | --- |
| KDM1A | 124 | 0.39 | 0.16-0.92 | 0.027 |
| KDM1B | 124 | 1.51 | 0.71-3.24 | 0.281 |
| KDM2A | 124 | 0.63 | 0.28-1.41 | 0.262 |
| KDM2B | 124 | 0.58 | 0.26-1.27 | 0.168 |
| KDM3A | 124 | 1.74 | 0.79-3.83 | 0.161 |
| KDM3B | 124 | 0.39 | 0.16-0.96 | 0.034 |
| JMJD1C | 124 | 0.69 | 0.30-1.58 | 0.373 |
| KDM4A | 124 | 1.31 | 0.61-2.78 | 0.488 |
| KDM4B | 124 | 0.55 | 0.22-1.38 | 0.198 |
| KDM4C | 124 | 9.82 | 1.33-72.44 | 0.006 |
| KDM4D | 124 | 0.51 | 0.20-1.27 | 0.139 |
| KDM4E | 124 | 0.34 | 0.16-0.74 | 0.005 |
| KDM5A | 124 | 0.49 | 0.21-1.11 | 0.081 |
| KDM5B | 124 | 0.47 | 0.22-1.00 | 0.045 |
| KDM5C | 124 | 1.88 | 0.79-4.44 | 0.145 |
| KDM5D | 124 | 0.34 | 0.13-0.90 | 0.022 |
| KDM6A | 124 | 2.27 | 0.99-5.20 | 0.046 |
| KDM6B | 124 | 0.52 | 0.24-1.14 | 0.098 |
| UTY | 124 | 0.38 | 0.15-1.02 | 0.045 |
| KDM7A | 124 | 1.67 | 0.78-3.59 | 0.181 |
| PHF8 | 124 | 0.63 | 0.30-1.35 | 0.231 |
| PHF2 | 124 | 0.40 | 0.14-1.15 | 0.078 |
| KDM8 | 124 | 0.43 | 0.15-1.23 | 0.105 |
| RIOX1 | 124 | 0.57 | 0.23-1.41 | 0.218 |
| RIOX2 | 124 | 0.67 | 0.31-1.44 | 0.300 |

**Supplementary Table S5:** **The relapse-free survival rates of all histone demethylases**

*Patients are divided based on best cutoff and follow-up thresholds with a 60-month follow-up period.

**Supplementary Materials and methods**

**protein purifications**

The gene of interest was subcloned into the pET28a vector (Novagen) and transformed into E. coli BL21(DE3) (Economic) cells for expression of the recombinant His-tagged protein. Bacterial cultures were grown at 37°C in Luria Broth (Miller’s LB Broth) to an OD600 of 0.8, after which protein expression was induced with IPTG (CyrusBioscience) and the cultures were incubated at 16°C overnight (~18 hours). Following centrifugation, the bacterial pellet was resuspended in binding buffer (50 mM HEPES, pH 7.4; 150 mM NaCl; 40 μM ZnSO₄) and lysed by sonication (Qsonica). The lysates were then clarified by high-speed centrifugation, and the resulting supernatant was incubated with Ni-NTA resin (IMAC Sepharose 6X Fast Flow) for affinity purification. The resin was sequentially washed with buffer containing increasing concentrations of imidazole (Affymetrix) and proteins were eluted with 250 mM imidazole. The eluate was initially concentrated and further purified by size-exclusion chromatography using FPLC (Cytiva, AKTA go) on a ProteoSEC 6–600 kDa column, yielding the final purified protein.

**Protein pull down assay**

The purified protein was incubated at a defined concentration with 10 μg of A/G magnetic beads (Millipore) and 1 μg of primary antibody in the previously described binding buffer at 4°C overnight (~18 hours). Following incubation, the beads were washed three times with IP wash buffer (137 mM NaCl, 2.7 mM KCl, 10 mM Na₂HPO₄, 1.8 mM KH₂PO₄, 0.1% Tween 20, pH 7.4). Protein complexes bound to the beads were eluted using IP lysis buffer at 85°C for 10 minutes. The eluted complexes were then analyzed by western blotting. In this experiment, 1 μg of KDM4C-∆N490 was used at a fixed concentration, while GATA1-∆C170 was titrated in a range of 0.0625–1 μg.
